# Systems Biology Elucidates Common Pathogenic Mechanisms between Nonalcoholic and Alcoholic-Fatty Liver Disease

**DOI:** 10.1371/journal.pone.0058895

**Published:** 2013-03-13

**Authors:** Silvia Sookoian, Carlos J. Pirola

**Affiliations:** 1 Department of Clinical and Molecular Hepatology, Institute of Medical Research-IDIM, University of Buenos Aires-National Council of Scientific and Technological Research (CONICET), Ciudad Autónoma de Buenos Aires, Argentina; 2 Department of Molecular Genetics and Biology of Complex Diseases, Institute of Medical Research-IDIM, University of Buenos Aires-National-Council of Scientific and Technological Research (CONICET), Ciudad Autónoma de Buenos Aires, Argentina; Michigan State University, United States of America

## Abstract

The abnormal accumulation of fat in the liver is often related either to metabolic risk factors associated with metabolic syndrome in the absence of alcohol consumption (nonalcoholic fatty liver disease, NAFLD) or to chronic alcohol consumption (alcoholic fatty liver disease, AFLD). Clinical and histological studies suggest that NAFLD and AFLD share pathogenic mechanisms. Nevertheless, current data are still inconclusive as to whether the underlying biological process and disease pathways of NAFLD and AFLD are alike. Our primary aim was to integrate *omics* and physiological data to answer the question of whether NAFLD and AFLD share molecular processes that lead to disease development. We also explored the extent to which insulin resistance (IR) is a distinctive feature of NAFLD. To answer these questions, we used systems biology approaches, such as gene enrichment analysis, protein–protein interaction networks, and gene prioritization, based on multi-level data extracted by computational data mining. We observed that the leading disease pathways associated with NAFLD did not significantly differ from those of AFLD. However, systems biology revealed the importance of each molecular process behind each of the two diseases, and dissected distinctive molecular NAFLD and AFLD-signatures. Comparative co-analysis of NAFLD and AFLD clarified the participation of NAFLD, but not AFLD, in cardiovascular disease, and showed that insulin signaling is impaired in fatty liver regardless of the noxa, but the putative regulatory mechanisms associated with NAFLD seem to encompass a complex network of genes and proteins, plausible of epigenetic modifications. Gene prioritization showed a cancer-related functional map that suggests that the fatty transformation of the liver tissue is regardless of the cause, an emerging mechanism of ubiquitous oncogenic activation. In conclusion, similar underlying disease mechanisms lead to NAFLD and AFLD, but specific ones depict a particular disease signature that has a different impact on the systemic context.

## Introduction

The abnormal accumulation of fat in the liver–or hepatic steatosis–is often related either to metabolic risk factors associated with metabolic syndrome (MetS) in the absence of alcohol consumption (nonalcoholic fatty liver disease, NAFLD) or to chronic alcohol consumption (alcoholic fatty liver disease, AFLD). Despite the fact that the causative *noxa* for each clinical entity is different, both the diseases share the same natural history; e.g., the evolution of liver histology of NAFLD and AFLD varies from simple steatosis to cirrhosis, including an increased risk of hepatocellular carcinoma [1], [2]. The clinical features are strengthened by the liver pathology as NAFLD and AFLD share a number of histological changes, including the presence of lobular inflammation, morphological changes in liver mitochondria, perivenular and perisinusoidal fibrosis, and even hepatocellular ballooning [3], [4]. In fact, nonalcoholic steatohepatitis (NASH) was initially regarded by Lugwig J et al. as a histological picture that mimics alcoholic hepatitis [5].

Interestingly, advances in genome analysis have shown that rs738409 C/G, a nonsynonymous coding (I148M) gene variant located in human patatin-like phospholipase domain containing 3 gene (*PNPLA3*, also known as adiponutrin), is critically involved in the genetic susceptibility of fatty liver in both NAFLD [6], [7] and AFLD [8]. Furthermore, rs738409 not only modulates the amount of intrahepatic triglyceride content, but also the histological disease severity, including necroinflammation and fibrosis in both NAFLD [7], [9] and AFLD [10], [11].

In addition, it has also been suggested that NAFLD and AFLD might have similar pathogenic mechanism because both are associated with hepatic inflammatory changes and local upregulation of cytokine production, along with increased fibrogenesis. Nevertheless, there is still inconclusive data regarding whether the underlying biological process and disease pathways of NAFLD and AFLD are identical; in fact, the differences between them have been suggested [12]. Consequently, some critical questions remain unanswered, such as whether insulin resistance (IR) is associated with both NAFLD and AFLD, whether hepatic necroinflammation is related to similar triggering events, and whether cardiovascular disease is equally associated with both the liver disorders.

The *omics* revolution (genomics, proteomis, transcriptomics, and metabolomics) has significantly changed our understanding about the pathogenesis of complex diseases.

Nevertheless, the multi-level high-throughput *omics* data are growing exponentially, sometimes negatively impacting our capacity of extracting and interpreting biological insights from them. Fortunately, interesting computational resources have been developed, such as data-mining techniques, which not only help us in assembling information from the biomedical literature, but are also devoted to uncover details that are of practical value for reveling disease pathogenesis.

Hence, our primary aim was to integrate genomic, molecular, and physiological data about NAFLD and AFLD to answer the question of whether both the diseases share the same underlying pathogenic mechanisms. In addition, we explored the biological processes and associated-disease pathways behind NAFLD and AFLD to evaluate the extent to which IR is (or is not) a distinctive feature between them.

To answer these questions, we used data mining and systems biology approaches, such as a gene enrichment analysis and protein–protein interaction networks.

## Materials and Methods

### Data collection and computational data mining

To assemble the available evidence about NAFLD- and AFLD-associated biological processes in a systematic manner, we used the text mining platform PESCADOR (Platform for Exploration of Significant Concepts Associated to co-Occurrence Relationships) [13]. This resource allows collecting information about NAFLD/AFLD-related pathobiology to predict further biomolecular interactions among genes and proteins associated with them. PESCADOR selects gene/protein co-occurrence pairs based on their relatedness to biological concepts, bringing together, under a common perspective, protein interactions that have not been studied under the same research focus [13].

Thus, with the queries “alcoholic AND (steatosis OR fatty liver) NOT (non or nonalcoholic)” for AFLD and “nonalcoholic OR non-alcoholic AND fatty liver OR steatosis” for NAFLD, we retrieved 823 papers and 1345 co-occurrences for AFLD, and 868 papers and 2217 co-occurrences for NAFLD. The query involves retrieving extensible markup language (XML) PubMed abstracts for PMID list, passing XML PubMed abstracts for NLPROT analysis (a tool for finding protein names in natural language text), and tagging protein names and performing co-occurrences analyses. After carrying out terms' tagging, a total of 228 gene/protein terms were identified for AFLD (**Table S1**) and 314 terms were found for NAFLD (**Table S2**).

Of note, the data mining method implemented in PESCADOR is based on the LAITOR (Literature Assistant for Identification of Terms co-Occurrences and Relationships) tool [14] that ensures the users the analysis of the meaning of the text, not just the presence of key words. Actually, LAITOR identifies biointeraction terms in the text of the abstracts according to a dictionary of biointeraction terms [13], [14]. In addition, other available platforms are not flexible enough as PESCADOR to filter interactions extracted from a PubMed query.

### Systems biology approaches for gene enrichment analysis and protein–protein interaction networks

Based on the list of genes/proteins identified as explained earlier, we decided to explore the interactions between them in an integrative fashion to provide a “functional molecular map” of both the clinical disorders. Thus, functional enrichment analysis was performed by the bioinformatic resource *ToppGene Suite* (http://toppgene.cchmc.org) and *ToppCluster* (http://toppcluster.cchmc.org), which could detect functional enrichment of the candidate genes/proteins list based on Transcriptome, Proteome, Regulome (TFBS and miRNA), Ontologies (gene ontology GO, Pathway), Phenotype (human disease and mouse phenotype), Pharmacome (Drug-Gene associations), literature co-citation, and other features [15].

This application was selected because the gene functional annotations are based on a comprehensive list of databases that includes among others, annotation for drugs, disease and mouse phenotype, miRNAs, and allows the identification and prioritization of novel disease candidate genes in the interactome. Furthermore, the statistical methods for quantitative enrichment are reliable [16].

In addition, we used a strategy of gene prioritization under the hypothesis that the already known associated disease loci and proteins might be useful as a template to look for unknown molecular targets involved in the pathogenesis of NAFLD and AFLD. Thus, we performed a comprehensive analysis of candidate regions generated by the freely accessible ENDEAVOUR software available at http://homes.esat.kuleuven.be/~bioiuser/endeavour/endeavour.php.

ENDEAVOUR is a software application for the computational prioritization of candidate genes underlying biological processes or diseases, based on their similarity to known genes involved in a disease as previously described [17]. The hypothesis of prioritization by ENDEAVOUR is that candidate test genes are ranked based on their similarity with a set of known training genes; this strategy allows expansion of the selection of putative molecular targets and prediction of new targets. Terms lists for NAFLD and AFLD, shown in **Table S1** and **Table S2**, were used as training genes in the Endeavour platform; subsequently, the application prioritized the entire human genome looking for similarities between the candidates and the models built with the training genes. This prioritization covers most of the aspects of knowledge available on genes and gene products (functional annotations, protein interactions, expression profiles, regulatory information, sequence-based data, and literature mining) [18].

This application was selected because its system was validated experimentally by extensive leave-one-out cross-validations showing an excellent performance [18]. In addition, the platform ENDEAVOUR allows users to prioritize candidate genes not only with respect to their biological processes but also diseases of interest. Furthermore, we have experimentally validated the putative associations of variants of *IGF1R* with arterial hypertension and *HNF4a* with type 2 diabetes after being predicted by this tool [19], [20].

Figures were constructed under the graphical platforms for exploring the biological networks, MEDUSA [21] or Cytoscape v2.8 [22].

## Results

**Figure 1:**
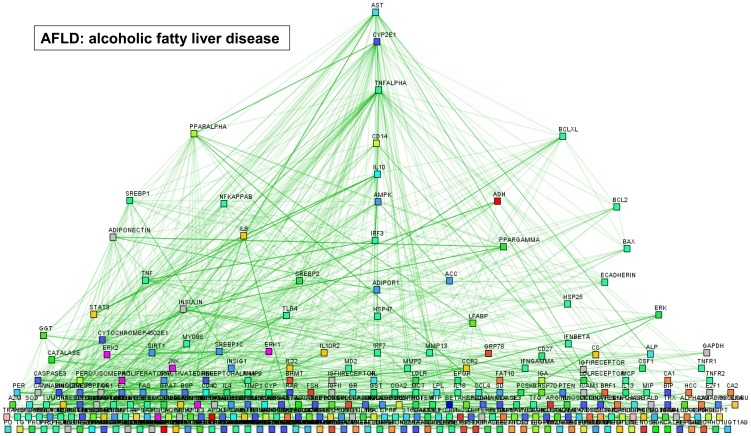
Graphic illustration of genes/proteins co-occurrence and their relatedness to biological concepts with the query “alcoholic AND (steatosis OR fatty liver) NOT (non or nonalcoholic)”. Prediction was performed by PESCADOR (available at http://cbdm.mdc-berlin.de/tools/pescador/), a web-based tool to assist large-scale integration text-mining of biointeractions extracted from MEDLINE abstracts. The graph was constructed using the free available program MEDUSA, which is a Java application for visualizing and manipulating graphs of interaction (www.bork.embl.de/medusa) [21], [33].

**Figure 2:**
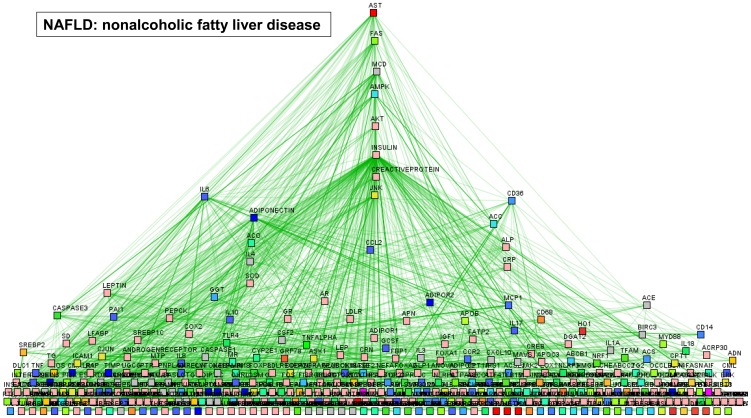
Graphic illustration of genes/proteins co-occurrence and their relatedness to biological concepts with the query “nonalcoholic OR non-alcoholic AND fatty liver OR steatosis”. Prediction was performed by PESCADOR (available at http://cbdm.mdc-berlin.de/tools/pescador/), a web-based tool to assist large-scale integration text-mining of biointeractions extracted from MEDLINE abstracts. The graph was constructed using the free available program MEDUSA, which is a Java application for visualizing and manipulating graphs of interaction (www.bork.embl.de/medusa) [21], [33].

The results of text mining for biomolecular interactions among terms associated with AFLD and NAFLD are shown in **Figures 1** and **2**, respectively.

Once AFLD-associated terms and interactions were displayed graphically, a dendriform hierarchical hub appeared centered on CYP2E1, TNFα, CD14 (a surface antigen that is preferentially expressed on monocytes/macrophages), IL10, PPARα, and the apoptosis regulator BCLXL (**Figure 1**). On the contrary, once NAFLD-associated terms were displayed graphically, a stepwise hierarchical central hub appeared centered on the apoptosis-mediating surface antigen FAS, the protein kinase AMP-activated AMPK (an energy sensor protein kinase that plays a key role in regulating cellular energy metabolism), insulin, and C-reactive protein (**Figure 2**); in addition, two-side nodes centered on IL6 and CD36 were also displayed.

Furthermore, based on the candidate gene/protein lists identified by the text-mining tool (**Table S1 and Table S2**), we performed a functional enrichment analysis. Interestingly, the analysis showed that AFLD-reported loci and proteins are integrated into several similar functional pathways and biological processes that did not significantly differ from those of NAFLD (**Table 1**). For example, analysis of GO molecular function showed that “receptor binding,” “cytokine receptor binding” and “lipid binding,” among other GO terms, were highly predicted for both AFLD and NAFLD. In fact, there was an almost perfect match between AFLD and NAFLD GO Molecular function terms, except for GO term “monocarboxylic acid binding” that was only predicted for AFLD (**Table 1**).

**Table 1:** Functional enrichment analysis of candidate genes and proteins previously associated with alcoholic liver disease (AFLD) and nonalcoholic fatty liver disease (NAFLD).

AFLD			NAFLD		
Id	Name/Source	P-value	Id	Name/Source	P-value
**GO: Molecular Function**					
GO:0005102	Receptor binding	2.238E-16	GO:0005102	Receptor binding	4.849E-25
GO:0042802	Identical protein binding	6.852E-14	GO:0046983	Protein dimerization activity	3.739E-23
GO:0005126	Cytokine receptor binding	8.920E-13	GO:0019899	Enzyme binding	5.795E-20
GO:0046983	Protein dimerization activity	8.254E-12	GO:0005126	Cytokine receptor binding	1.882E-15
GO:0019899	Enzyme binding	9.866E-12	GO:0042802	Identical protein binding	5.811E-15
GO:0008289	Lipid binding	3.766E-8	GO:0005125	Cytokine activity	1.539E-12
GO:0005125	Cytokine activity	7.216E-8	GO:0042562	Hormone binding	1.109E-11
GO:0033293	Monocarboxylic acid binding	9.596E-8	GO:0042803	Protein homodimerization activity	2.689E-11
GO:0042803	Protein homodimerization activity	3.205E-7	GO:0008289	Lipid binding	1.859E-9
GO:0031406	Carboxylic acid binding	1.372E-8	GO:0043565	Sequence-specific DNA binding	2.574E-8
**GO: Biological Process**					
GO:0010033	Response to organic substance	2.582E-64	GO:0010033	Response to organic substance	7.690E-75
GO:0002376	Immune system process	7.621E-41	GO:0009719	Response to endogenous stimulus	7.330E-53
GO:0009719	Response to endogenous stimulus	1.071E-39	GO:0009725	Response to hormone stimulus	2.511E-51
GO:0009611	Response to wounding	4.116E-39	GO:0070887	Cellular response to chemical stimulus	1.366E-48
GO:0048583	Regulation of response to stimulus	6.783E-39	GO:0009893	Positive regulation of metabolic process	3.162E-45
GO:0002682	Regulation of immune system process	1.175E-37	GO:0010941	Regulation of cell death	1.318E-44
GO:0009725	Response to hormone stimulus	4.058E-37	GO:0006629	Lipid metabolic process	4.993E-44
GO:0006955	Immune response	1.572E-35	GO:0042981	Regulation of apoptotic process	4.620E-43
GO:0051704	Multi-organism process	4.325E-35	GO:0009605	Response to external stimulus	5.391E-43
GO:0009605	Response to external stimulus	2.079E-33	GO:0043067	Regulation of programmed cell death	1.205E-42
**Pathway**					
hsa04620	Toll-like receptor signaling pathway	7.303E-15	hsa04920	Adipocytokine signaling pathway	6.346E-17
reg_gr_pathway	Glucocorticoid receptor regulatory network	3.032E-10	hsa05200	Pathways in cancer	4.055E-14
hsa05200	Pathways in cancer	3.075E-10	P00006	Apoptosis signaling pathway	2.392E-13
P00054	Toll receptor signaling pathway	1.569E-8	hsa04210	Apoptosis	2.074E-11
BIOCARTA_PPARA_PATHWAY	Mechanism of Gene Regulation by Peroxisome Proliferators via PPARa(alpha)	1.958E-8	hsa04620	Toll-like receptor signaling pathway	4.204E-11
BIOCARTA_IL1R_PATHWAY	Signal transduction through IL1R	2.473E-8	P00036	Interleukin signaling pathway	1.550E-10
BIOCARTA_CYTOKINE_PATHWAY	Cytokine Network	4.812E-8	hsa05215	Prostate cancer	2.758E-10
P00006	Apoptosis signaling pathway	5.503E-8	nfat_tfpathway	Calcineurin-regulated NFAT-dependent transcription in lymphocytes	3.064E-9
hsa04060	Cytokine-cytokine receptor interaction	5.858E-8	BIOCARTA_KERATINOCYTE_PATHWAY	Keratinocyte Differentiation	2.183E-8
BIOCARTA_INFLAM_PATHWAY	Cytokines and Inflammatory Response	1.021E-7	hsa05014	Amyotrophic lateral sclerosis (ALS)	
**Gene family**					
CD	CD molecules	1.028E-9	CD	CD molecules	9.160E-11
IL	Interleukins and interleukin receptors	4.929E-7	IL	Interleukins and interleukin receptors	2.368E-8
CA	Carbonic anhydrases	4.284E-4	ABC	ATP-binding cassette transporters	1.523E-5
			CASP	Caspases	5.740E-5
			ACS	Acyl-CoA synthetases	4.506E-2
**Interaction**					
int:UBC	UBC interactions	4.915E-10	int:UBC	UBC interactions	5.220E-15
int:SP1	SP1 interactions	5.599E-10	int:NCOR2	NCOR2 interactions	2.639E-11
int:MAPK1	MAPK1 interactions	6.431E-6	int:STAT3	STAT3 interactions	2.315E-9
int:CAV1	CAV1 interactions	1.121E-5	int:IRS1	IRS1 interactions	9.739E-9
int:MAPK8	MAPK8 interactions	1.441E-5	int:EP300	EP300 interactions	9.902E-9
**Mouse phenotype**					
MP:0005370	Liver/biliary system phenotype	1.042E-24	MP:0002118	Abnormal lipid homeostasis	8.401E-36
MP:0000598	Abnormal liver morphology	2.556E-23	MP:0001547	Abnormal lipid level	5.444E-35
MP:0002138	Abnormal hepatobiliary system morphology	8.746E-23	MP:0000187	Abnormal triglyceride level	1.208E-34
MP:0002118	Abnormal lipid homeostasis	1.195E-19	MP:0003949	Abnormal circulating lipid level	4.153E-34
MP:0001547	Abnormal lipid level	1.766E-19	MP:0000188	Abnormal circulating glucose level	8.678E-32

The analysis was done by the bioinformatic resource *ToppGene Suite*. Table 1 shows only top ranked and highly significant association. GO: gene ontology (http://www.geneontology.org/).

Functional analysis of the top 10 GO Biological Process showed that AFLD was significantly associated with the modulation of immune function because the highly significant terms were GO:0002376 Immune system process, GO:0002682 Regulation of immune system process, and GO:0006955 Immune response (**Table 1**). In contrast, functional analysis of the top 10 GO Biological Process showed that NAFLD was significantly associated with GO:0009725 Response to hormone stimulus, GO:0009893 Positive regulation of metabolic process, GO:0006629 Lipid metabolic process, and GO:0042981 Regulation of apoptotic process.

Accordingly, AFLD was significantly associated with toll-like receptor signaling and cytokine related pathways, while NAFLD showed significant association with adipocytokine and apoptosis signaling pathway (**Table 1**). It is worth mentioning that pathways in cancer were highly predicted for both the diseases, and keratinocyte differentiation was a distinctive pathway only predicted for NAFLD.

Among the highly ranked gene families, CD (cluster of differentiation) molecules as well as interleukins and interleukin receptors were overrepresented in both AFLD and NAFLD, but ATP-binding cassette transporters, caspases, and acyl-CoA synthetases were only significantly predicted in NAFLD (**Table 1**). Remarkably, predicted interactions among candidate terms for AFLD showed mitogen-activated protein kinases as highly ranked. Conversely, STAT3, IRS1, NCOR2 (a transcriptional co-repressor of NR4A2/NURR1 that acts through histone deacetylases, HDACs), and EP300 (E1A-binding protein p300 that functions as HDAC regulating transcription via chromatin remodeling during cell proliferation and differentiation) were significant for NAFLD (**Table 1**), suggesting, as we recently reported, that epigenetic factors play a critical role in the disease progression, not only involving nuclear DNA [23], but mitochondrial DNA as well [24].

Prediction of relatedness with mouse phenotype showed that candidate AFLD genes/proteins were significantly associated with abnormal liver and hepatobiliary system morphology, and to a lesser extent with lipid metabolism; on the other hand, candidate NAFLD gene/ proteins were significantly associated with glucose and lipid homeostasis (**Table 1**).

**Figure 3:**
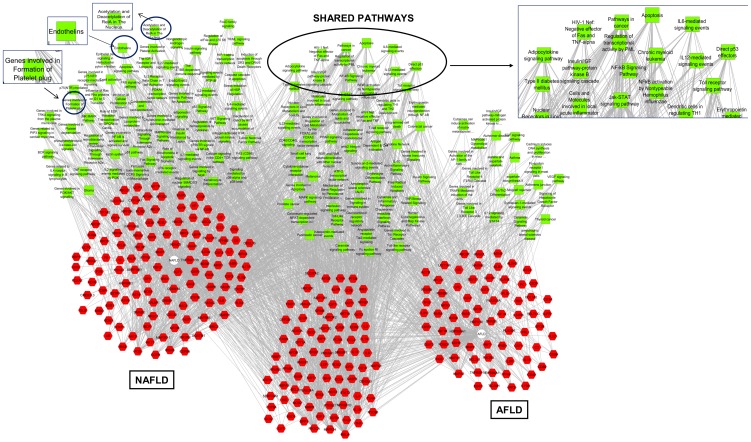
Graphic illustration of a functional modular map of the multiple gene/ protein analysis encompassing the candidate list of NAFLD and AFLD based on disease pathways. Results of functional association analysis performed by the bioinformatics resource *ToppCluster* (http://toppcluster.cchmc.org) based on pathways networks showing enriched terms from Gene Ontology, Mouse Phenotype, Co-expression, microRNAs, and transcription factors for the NAFLD- and AFLD-specific gene/protein lists. Right side of the figure depicts the highly significant enrichments for sets of genes and proteins of the NAFLD term list; left side of the figure depicts the highly significant enrichments for sets of genes and proteins of the AFLD term list; and the analysis of genes and intersection of pathways between NAFLD and AFLD is shown in the center part of the figure. Terms in red represent genes/proteins, and terms in green represent disease pathways in GO terms. The graph was constructed using the free available program Cytoscape, a software project for integrating biomolecular interaction networks with high-throughput expression data and other molecular states into a unified conceptual framework [22].

We further performed a multiple genes/ proteins analysis that simultaneously encompassed the candidate list of AFLD and NAFLD to obtain a functional modular map that identifies shared and specific features of each disease, including potential regulatory mechanism relationships. **Figure 3** summarizes the disease pathways shared by AFLD and NAFLD, and those that are overrepresented specifically for each disease. Remarkably, apoptosis, IL6-mediated signaling, NF-κ-β and JAK-STAT pathways, metabolism of lipids and lipoproteins, and pathways in cancer were placed in the overlapping zone and were shared between the two data sets. Accordingly, interleukins (1A, 3, 4, 10, and 18), adiponectin, PPARs (α and γ), sirtuin 1, TNFα, STAT3, INSR, SERBP1 or PAI-1, ICAM1, among the others, were also shared between the two sets (**Figure 3**).

Cross-comparing enrichment analyses showed that NAFLD is associated with a myriad of complex pathways that include among the others, insulin signaling, caspases and mitochondrial-related apoptosis, stress induction of heat shock proteins, cellular proliferation, hypoxia induction, and protein associated with epigenetic regulation (**Figure 3**).

Conversely, cross-comparing enrichment analyses showed that AFLD is associated with a more reduced network of disease pathways, mostly focused on modulation of the immune response, toll-like receptor signaling, and cytokines (**Figure 3**).

Interestingly, cardiovascular-related pathways were more enriched in NAFLD in comparison with AFLD (platelet plug formation and endothelins), as shown in **Figure 3**. Regarding lipid metabolism, AFLD pathways were associated with glycerolipids and ceramid signaling, and NAFLD pathways were associated with lipoprotein metabolism and chylomicron-mediated lipoprotein transport (**Figure 3**). Details about clusters of function-disease-related genes and proteins are given in **Figure S1**, and a functional modular map of the multiple gene/ protein analysis encompassing the candidate list of NAFLD and AFLD based on cellular component is shown in **Figure S2**. Of note, although there are specific genes associated with either AFLD or NAFLD, there is not a specific pathway for both of the diseases.

### Is alcohol associated with impaired insulin signaling and IR?

Our secondary aim was to explore whether impaired insulin signaling is a molecular process associated with both the conditions, regardless of the insult (either metabolic or alcohol), or if IR is restricted to NAFLD and thereby to MetS.

**Figure 4:**
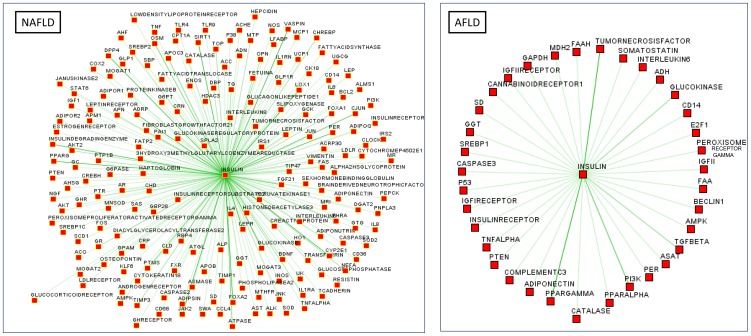
Comparative co-analysis of NAFLD and AFLD data sets focused on insulin signaling. Results of functional association analysis performed by the bioinformatics resource PESCADOR (available at http://cbdm.mdc-berlin.de/tools/pescador/), a web-based tool to assist large-scale integration text-mining of biointeractions extracted from MEDLINE abstracts with a focus in the selected terms. The graph was constructed using the free available program, MEDUSA, which is a Java application for visualizing and manipulating graphs of interaction (www.bork.embl.de/medusa) [21], [33]. The thickness of the green lines signifies greater significance.

Comparative co-analysis of both the data sets showed that insulin signaling is impaired in both the liver disorders, but the biological processes and putative regulatory mechanisms associated with NAFLD seem to encompass a large interconnected network of genes and proteins, including, but not restricted to, FOXO1, SIRT1, dipeptidylpeptidase IV (DPP4, a cell surface glycoprotein receptor involved in the co-stimulatory signal essential for T-cell receptor-mediated T-cell activation and also an enzyme involved in neuropeptides and incretins degradation, i.e. NPY and GLP1), PPARγ, PER, and HDAC3 (**Figure 4**). By the contrary, insulin signaling in AFLD is highly related to and specifically focused on TNFα, PPARγ, and TGBFβ (**Figure 4**).

Gene prioritizations shows that fatty liver either associated with metabolic factors or alcohol is strongly associated with cancer pathways and ubiquitously malignant cellular transformation

We used the above-explained approach of gene prioritization, and based on the results of ENDEAVOUR output, we selected the top-ranked candidate genes prioritized for NAFLD (**Table S3**) and AFLD (**Table S4**), based on the top-ranked significant *P-values* that reflect the significance of enrichment. Then, we performed a new comparative co-analysis of both the data sets generated by ENDEAVOUR, and we explored novel putative disease pathways. Surprisingly, in addition to genes associated with IR or type 2 diabetes, i.e. HNF4A [20], the integrative functional analysis of the shared pathways between NAFLD and AFLD showed a significant enrichment of biological processes associated with cancer and neoplastic transformation of other nonhepatic tissues, such as colorectal, prostate, pancreas, bladder, renal, endometrial, and thyroid cancer (**Figure S3**). Moreover, growth factors associated signaling pathways involved in cell proliferation, cell migration, survival, and chemotaxis, such as IGF, PDGF or their receptors, and growth factors active in angiogenesis and endothelial cell growth, such as VEGF, were also highly enriched (**Figure S3**).

### Diseases-related microRNAs and regulation of gene transcription in AFLD and NAFLD

**Figure 5:**
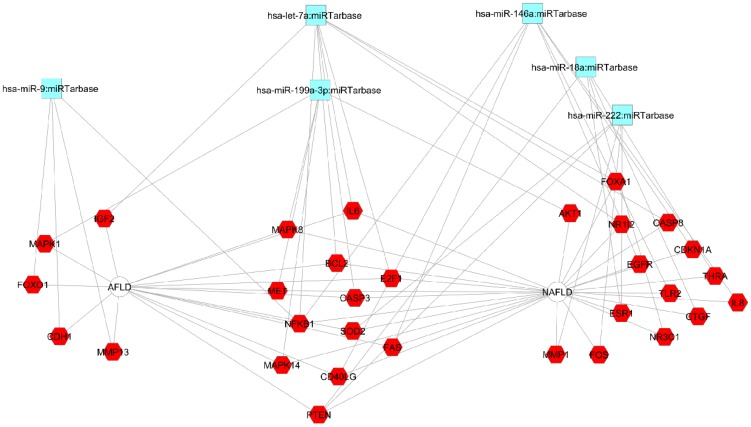
Functional enrichment analysis of putative miRNAs associated with NAFLD and AFLD. The network is shown as a cytoscape graph [22] generated from *ToppCluster* (available at toppcluster.cchmc.org/) network analysis.

Integrative functional analysis showed novel microRNAs (miRNAs) that might modulate AFLD, such as miR-9, or NALFD, such as miR-146a, miR-18a, and miR-22 (**Figure 5**). Remarkably, miR-7a and miR-199a-3p were found to be placed in the shared area of AFLD and NAFLD, and were predicted as related to apoptosis and inflammation-related network integrated by IL6, BCL2, caspase 3, NF-κ-β, CD40L, MAPKs, and PTEN (**Figure 5**). Again, it was noted that there is no specific miRNA associated with a gene involved in any particular disease.

Comparative co-analysis of both the data sets generated by ENDEAVOUR platform showed an even more complex prediction of miRNA (**Figure S4**). Interestingly, based on these different targets, some miRNAs, such as miR-7a and miR-146a, were once again predicted.

## Discussion

NAFLD and AFLD are both leading causes of non-viral chronic liver diseases, and the prevalence of these two clinical disorders is constantly growing worldwide.

There is agreement about the fact that the clinical distinction between NAFLD and AFLD is often challenging, and misclassification owing to difficulties in collecting reliable information about the amount of alcohol consumption from patients is a source of bias. Likewise, the pathogenic distinction between both the disorders is not only fuzzy, but physicians agree that similar major molecular mechanisms are shared between NAFLD and AFLD, including inflammatory pathways and fibrogenesis [12], [25]. Nevertheless, the evidence is still questionable because the disease pathways and molecular process are often explored individually, instead of working in concert, and these liver disorders are seldom treated as what they truly are: complex diseases. In this study, we proposed an exploration about the disease pathways associated with NAFLD and AFLD based on systems biology under the hypothesis that a more integrative analysis of the pathogenesis of these diseases may have a strong impact not only on their clinical and molecular knowledge, but also on the interventional programs and putative emerging therapies. In that sense, our analysis showed that vitamins (vitamin A, C, D, and E), natural substances (resveratrol, genistein, hormones), or drugs (metformin, statins, indomethacin) may have beneficial effects on fatty liver, independently of the causative noxa (**Figure S5**).

### What this study tells us about the pathogenesis of NAFLD and AFLD?

Our primary result basically showed that the leading biological process and disease pathways associated with NAFLD did not significantly differ from those predicted for AFLD. Nevertheless, systems biology revealed the weight of each molecular process behind each of the two diseases, and dissected distinctive molecular NAFLD and AFLD signatures.

For instance, apoptosis seems to be the common cell death process in NAFLD and AFLD, but AFLD is mainly associated with the so-called extrinsic pathway related to tumor necrosis family, which would be modulated by proapoptotic BCl2 family members. In contrast, NAFLD seems to be primary associated with FAS-induced apoptosis, which is highly interconnected to an intricate network of metabolic stressors, activation of caspases, and a collection of proteins that modulate apoptosis, necrosis, and inflammation. It is worth mentioning that changes in mitochondrial membrane permeabilization and endoplasmic reticulum stress are central features in both AFLD and NAFLD, as shown in the prediction of the cellular compartment (**Figure S2**).

Moreover, integration of the biological process and disease pathways associated with NAFLD showed that the fatty liver of the MetS reprograms the body lipid and glucose metabolism, and these events might be mediated, among the others, by hypoxia and epigenetic changes. The latter observation is in agreement with previous novel findings of our group, which demonstrated that DNA methylation of gene promoters in the liver tissue is critically involved in the modulation of peripheral IR [23], and epigenetic changes in mitochondrial DNA mediates NAFLD disease progression [24]. Furthermore, the integrative analysis focused on interactions among terms significantly predicted histone acetyltransferase p300 (EP300), which is a co-activator of hypoxia-inducible factor 1α and is an acetyltransferase for histone and nonhistone targets, all of which are highly involved in the endogenous circadian clock pathway. It is noteworthy that *CLOCK* variants were reported to be associated with NAFLD [26].

NAFLD was also found to be associated with keratinocyte differentiation pathway, which suggests the involvement of MAPK pathway in the disease biology; this finding is plausible with the molecular cascades involved in apoptosis, inflammation, cell growth, and differentiation observed in this disease.

Conversely, integration of the biological process and disease pathways associated with AFLD showed that the insult triggers a strong local immune response that is associated with the release of a plethora of cytokines. Thereby, all further metabolic changes in the liver tissue are downstream events of the local and powerful immune response.

Some comments about the limitations of this study may be added. For example, despite the fact that NAFLD and AFLD are processes that have been studied for decades, some unpublished aspects yet to be uncovered were not included in data mining. Hence, the results of this study only integrate the published knowledge about both the diseases. Nevertheless, although the functional exploration and enrichment analysis have largely expanded the pre-existing knowledge, the term list of each disease has the limitations of any method that relies on literature analysis.

### What this study tells us about the impact of NAFLD and AFLD on the risk of systemic diseases?

Functional enrichment analysis showed interesting areas of putative future research, such as the role of the predicted miRNAs–let-7a, miR-146a, and miR-199a–in the pathogenesis of both the diseases. Remarkably, miR-199a has been recently found to be involved in myocardial infarction and other cardiac diseases [27], and miRNA-let-7a has been noted to be involved in epigenetics-miRNA regulatory pathways [28]. These findings remain unexplored and deserve follow-up and exploration in human studies, particularly because we do believe that miRNAs may constitute a novel endocrine system [24].

Although there are numerous studies showing molecular mechanisms associated with NAFLD and AFLD, they have not explained how molecular mediators interact with each other and how these interactions perturb the systemic homeostasis. In this study, we showed that both NAFLD and AFLD are strongly associated with cancer-related pathways that do not seem to be restricted to the liver. Surprisingly, comparative co-analysis of NAFLD- and AFLD- related biological terms showed a cancer-related functional map that suggests that the fatty transformation of liver tissue, regardless of the insult, is an emerging mechanism of oncogenic activation. These findings are supported by previous clinical observations [29]–[31]. Moreover, our data may explain previous reports about NAFLD patients predisposed to hepatocellular carcinoma in the absence of cirrhosis [32]. It would be interesting to answer this question in clinical studies that explore for instance, patterns of gene expression in hepatocellular carcinoma and surrounding nonneoplastic liver tissue in noncirrhotic patients with NAFLD and AFLD.

Finally, two remarkable findings were emphasized by this study. First, systems biology shed light on the participation of NAFLD, but not AFLD, in cardiovascular disease, because the integrative analysis highlighted the role of NAFLD in thrombotic events and modulation of vasculature behavior by the release of endothelins. Second, NAFLD and AFLD were found to be associated with impairment of insulin signaling and IR; while NAFLD-linked IR was noted to be a multifaceted process that involves several molecular processes working in concert; on the other hand, AFLD-linked IR was observed to be rather the consequence of TNFα-related signaling and subsequent local modulation of the insulin receptor-activated pathways.

In conclusion, over the past 40 years many advances have been made in our understanding of fatty liver and the mechanisms by which it develops. New evidence from the clinical classification of NAFLD and AFLD suggests that there are shared mechanisms between them. Hence, taken together, these data suggest that similar disease mechanisms lead to the clinical outcome of NAFLD and AFLD, but specific ones depict a particular signature that correlates to the impact of each phenotype in the systemic context. The molecular understanding of the shared and specific mechanisms will improve our knowledge of how fatty liver and disease progression occur, eventually leading to the development of improved noninvasive diagnostic tools and novel therapeutic agents.

For instance, the analysis of putative drugs associated with the explored disease pathways might suggest that some natural substances, like resveratrol, or some drugs like metformin, losartan or statins, might be equally beneficial to improve fatty liver, independently of the causative noxa. On the other hand, in order to envision non invasive diagnostic tools for monitoring the disease severity, one might speculate that pathways associated with TNFα-mediated immune response might be useful for AFLD and pathways associated with FAS-induced apoptosis and caspases activation might be effective for NAFLD.

Likewise, this knowledge would help to relax our restrictions on NAFLD/AFLD disease classification based specifically on the amount of alcohol consumption when limits are not toxic quantities, but barely exceed the NAFLD classification limits (∼60–120 g/day of alcohol).

### Implications and future directions

An integrative knowledge about the disease pathogenesis of NAFLD and ALFD will pave the way towards formulation of new hypothesis. In addition, if underlying mechanisms are common, the same future therapeutic approaches may improve both conditions. Nevertheless, more experimental data and clinical studies are needed in order to accomplish this observation.

## Supporting Information

Figure S1 Graphic illustration of **a functional modular map of the multiple gene/ protein analysis encompassing the candidate list of NAFLD and AFLD based on functional genes.** Results of functional association analysis performed by the bioinformatics resource *ToppCluster* (http://toppcluster.cchmc.org). Right side of the figure depicts the highly significant enrichments for sets of genes (red hexagon) of the NAFLD term list; left side of the figure depicts the highly significant enrichments for sets of genes of the AFLD term list; and the analysis of the intersection of genes and gene functions (green squares) between NAFLD and AFLD is shown in the center of the figure. The network is shown as a cytoscape graph.
(TIF)

Figure S2 Graphic illustration of **a functional modular map of the multiple gene/ protein analysis encompassing the candidate list of NAFLD and AFLD based on cellular component.** Results of functional association analysis performed by the bioinformatics resource *ToppCluster* (http://toppcluster.cchmc.org). Right side of the figure depicts the highly significant enrichments for cellular components (green squares) of the NAFLD-term list; left side of the figure depicts the highly significant enrichments for cellular components of the AFLD term list; and the genes (red hexagons) and analysis of the intersection between NAFLD and AFLD is shown in the center of the figure. The network is shown as a cytoscape graph.
(TIF)

Figure S3 **Computational prioritization of candidate genes underlying NAFLD and AFLD and comparative co-analysis of genes pathways (green squares).** Prioritization was done by the bioinformatic tool ENDEAVOUR, and the figure shows the results of the cluster analysis of the first top 200 prioritized candidate genes from the whole human genome (23.712 genes), with a significant association with the training set of NAFLD and AFLD. Functional association analysis was performed by the bioinformatics resource *ToppCluster* (http://toppcluster.cchmc.org). Right side of the figure depicts the highly significant enrichments for sets of genes (red hexagons) of the NAFLD term list; left side of the figure depicts the highly significant enrichments for sets of genes of the AFLD term list; and the analysis of the intersection of functional genes between NAFLD and AFLD is shown in the center of the figure. The network is shown as a cytoscape graph.
(TIF)

Figure S4 **Computational prioritization of candidate genes underlying NAFLD and AFLD and comparative co-analysis of predicted miRNAs (violet squares).** Prioritization was done by the bioinformatic tool ENDEAVOUR, and the figure shows the results of the cluster analysis of the first top 200 prioritized candidate genes (red squares) from the whole human genome (23.712 genes), with a significant association with the training set of NAFLD and AFLD. Functional association analysis was performed by the bioinformatics resource *ToppCluster* (http://toppcluster.cchmc.org). The network is shown as a cytoscape graph.
(TIF)

Figure S5 **Computational prioritization of candidate genes underlying NAFLD and AFLD and comparative co-analysis of predicted drugs (orange squares).** Prioritization was done by the bioinformatic tool ENDEAVOUR, and the figure shows the results of the cluster analysis of the first top 200 prioritized candidate genes (red squares) from the whole human genome (23.712 genes), with a significant association with the training set of NAFLD and AFLD. Functional association analysis was performed by the bioinformatics resource *ToppCluster* (http://toppcluster.cchmc.org). The network is shown as a cytoscape graph.
(TIF)

Table S1 **Genes terms identified in 823 published abstracts by the PESCADOR platform (**P**latform for** E**xploration of** S**ignificant** C**oncepts** A**ssociated** **to co-**O**ccurrence** R**elationships) with the query “alcoholic AND (steatosis OR fatty liver) NOT (non or nonalcoholic)” for AFLD.**
(DOC)

Table S2 **Genes terms identified in 868 published abstracts by the PESCADOR platform (**P**latform for** E**xploration of** S**ignificant** C**oncepts** A**ssociated** **to co-**O**ccurrence** R**elationships) with the query “nonalcoholic OR non-alcoholic) AND (fatty liver OR steatosis)” for NAFLD**.
(DOC)

Table S3 **Results of gene prioritization: the top ranked candidate genes prioritized for NAFLD.**
(DOC)

Table S4 **Results of gene prioritization: the top ranked candidate genes prioritized for AFLD.**
(DOC)
