# Supplementary figures and images for: Systems Biology Elucidates Common Pathogenic Mechanisms between Nonalcoholic and Alcoholic-Fatty Liver Disease
Source: PLoS One. 2013 Mar 13;8(3):e58895. doi: 10.1371/journal.pone.0058895 (PMC3596348; doi:10.1371/journal.pone.0058895)

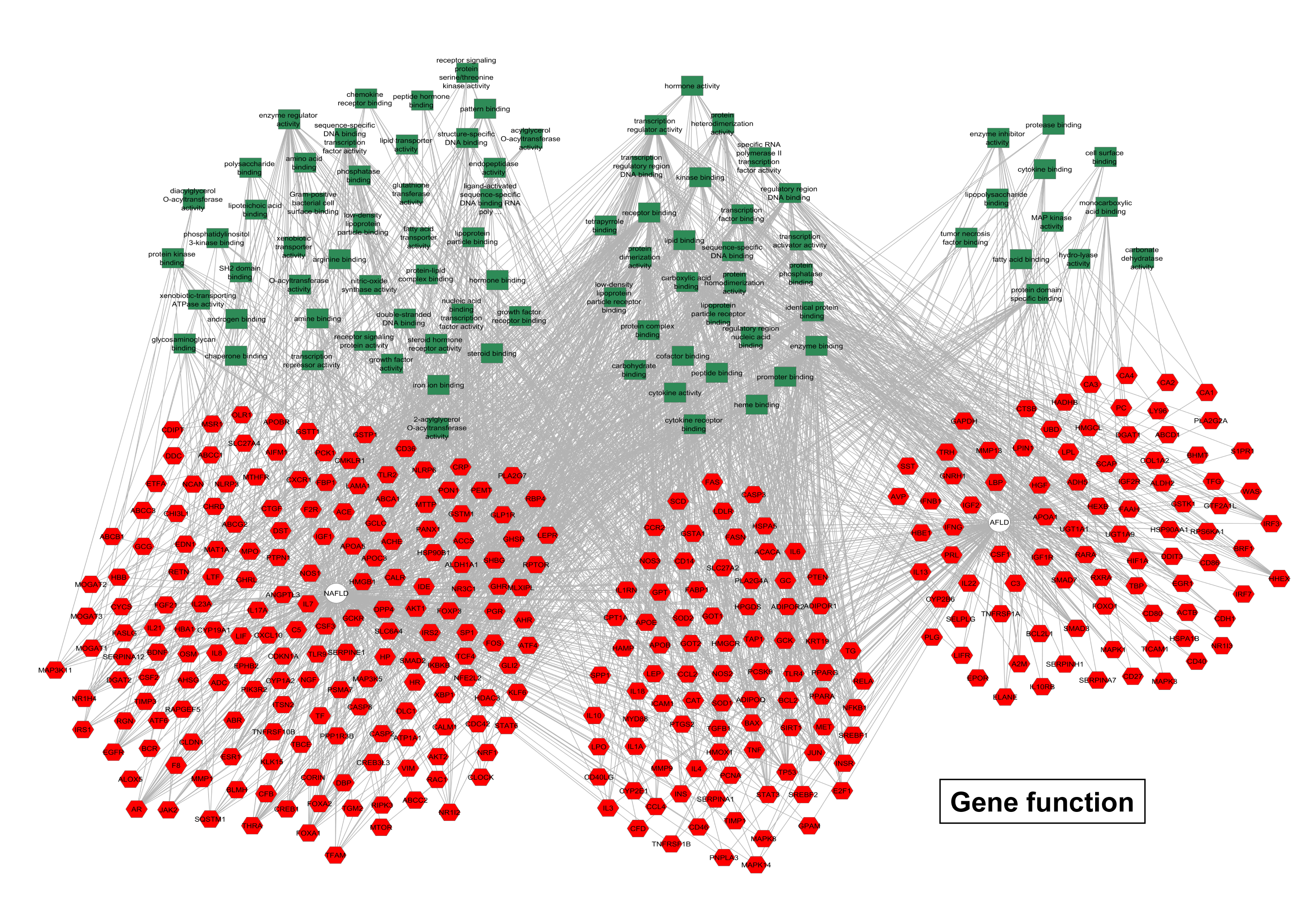

Supplement: Figure S1 — Graphic illustration of a functional modular map of the multiple gene/ protein analysis encompassing the candidate list of NAFLD and AFLD based on functional genes. Results of functional association analysis performed by the bioinformatics resource ToppCluster (http://toppcluster.cchmc.org). Right side of the figure depicts the highly significant enrichments for sets of genes (red hexagon) of the NAFLD term list; left side of the figure depicts the highly significant enrichments for sets of genes of the AFLD term list; and the analysis of the intersection of genes and gene functions (green squares) between NAFLD and AFLD is shown in the center of the figure. The network is shown as a cytoscape graph. (TIF) [file pone.0058895.s001.tif]

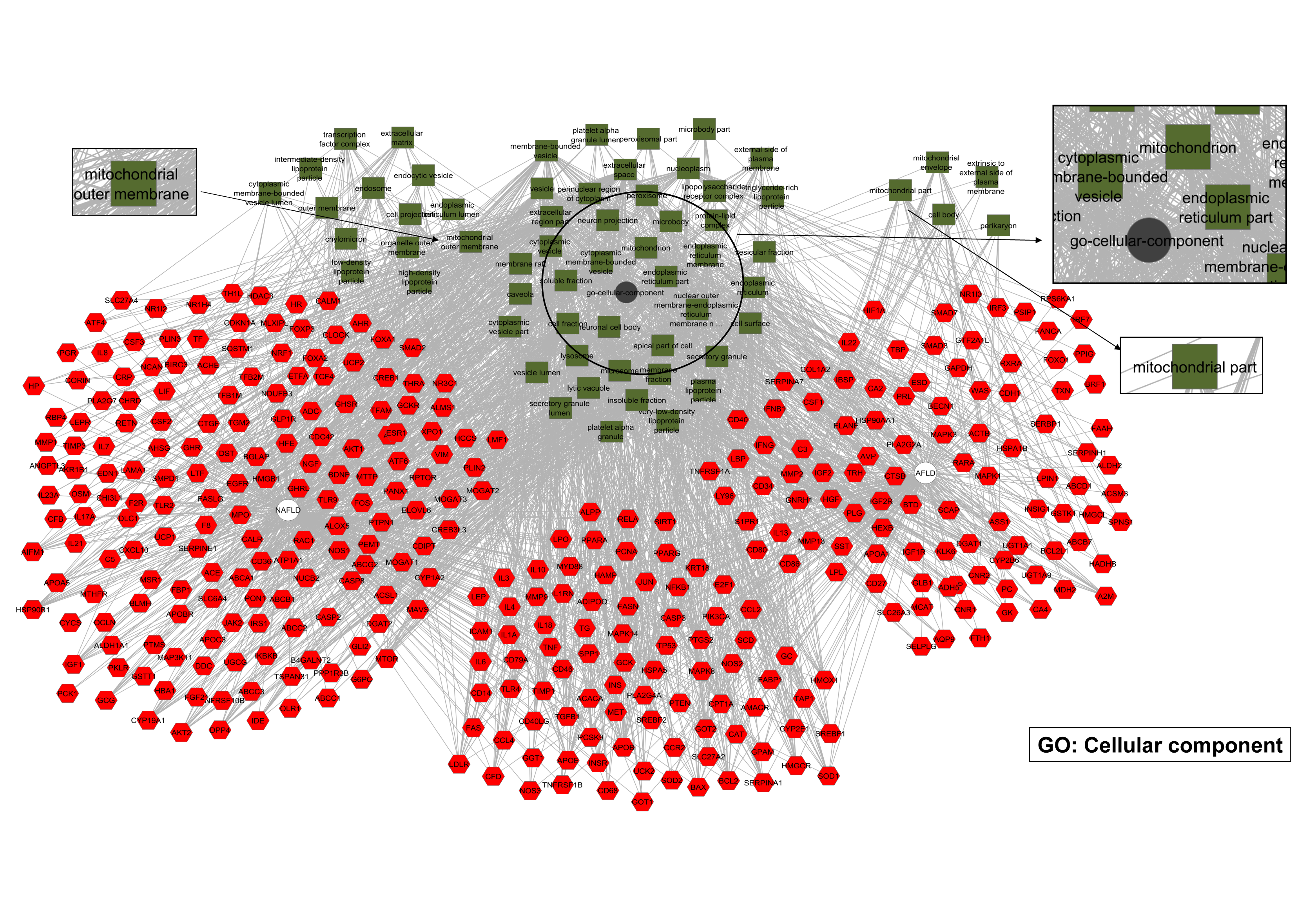

Supplement: Figure S2 — Graphic illustration of a functional modular map of the multiple gene/ protein analysis encompassing the candidate list of NAFLD and AFLD based on cellular component. Results of functional association analysis performed by the bioinformatics resource ToppCluster (http://toppcluster.cchmc.org). Right side of the figure depicts the highly significant enrichments for cellular components (green squares) of the NAFLD-term list; left side of the figure depicts the highly significant enrichments for cellular components of the AFLD term list; and the genes (red hexagons) and analysis of the intersection between NAFLD and AFLD is shown in the center of the figure. The network is shown as a cytoscape graph. (TIF) [file pone.0058895.s002.tif]

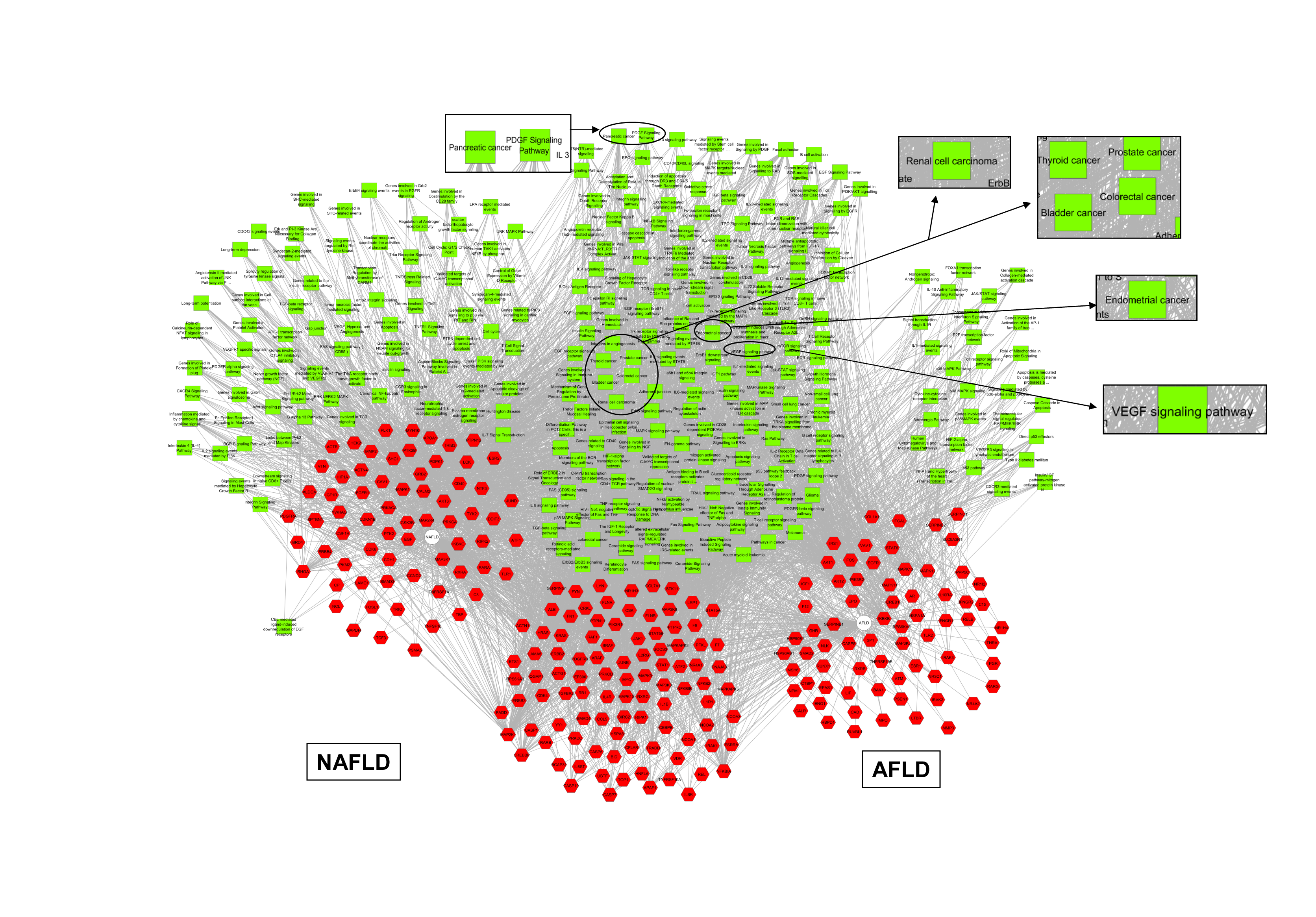

Supplement: Figure S3 — Computational prioritization of candidate genes underlying NAFLD and AFLD and comparative co-analysis of genes pathways (green squares). Prioritization was done by the bioinformatic tool ENDEAVOUR, and the figure shows the results of the cluster analysis of the first top 200 prioritized candidate genes from the whole human genome (23.712 genes), with a significant association with the training set of NAFLD and AFLD. Functional association analysis was performed by the bioinformatics resource ToppCluster (http://toppcluster.cchmc.org). Right side of the figure depicts the highly significant enrichments for sets of genes (red hexagons) of the NAFLD term list; left side of the figure depicts the highly significant enrichments for sets of genes of the AFLD term list; and the analysis of the intersection of functional genes between NAFLD and AFLD is shown in the center of the figure. The network is shown as a cytoscape graph. (TIF) [file pone.0058895.s003.tif]

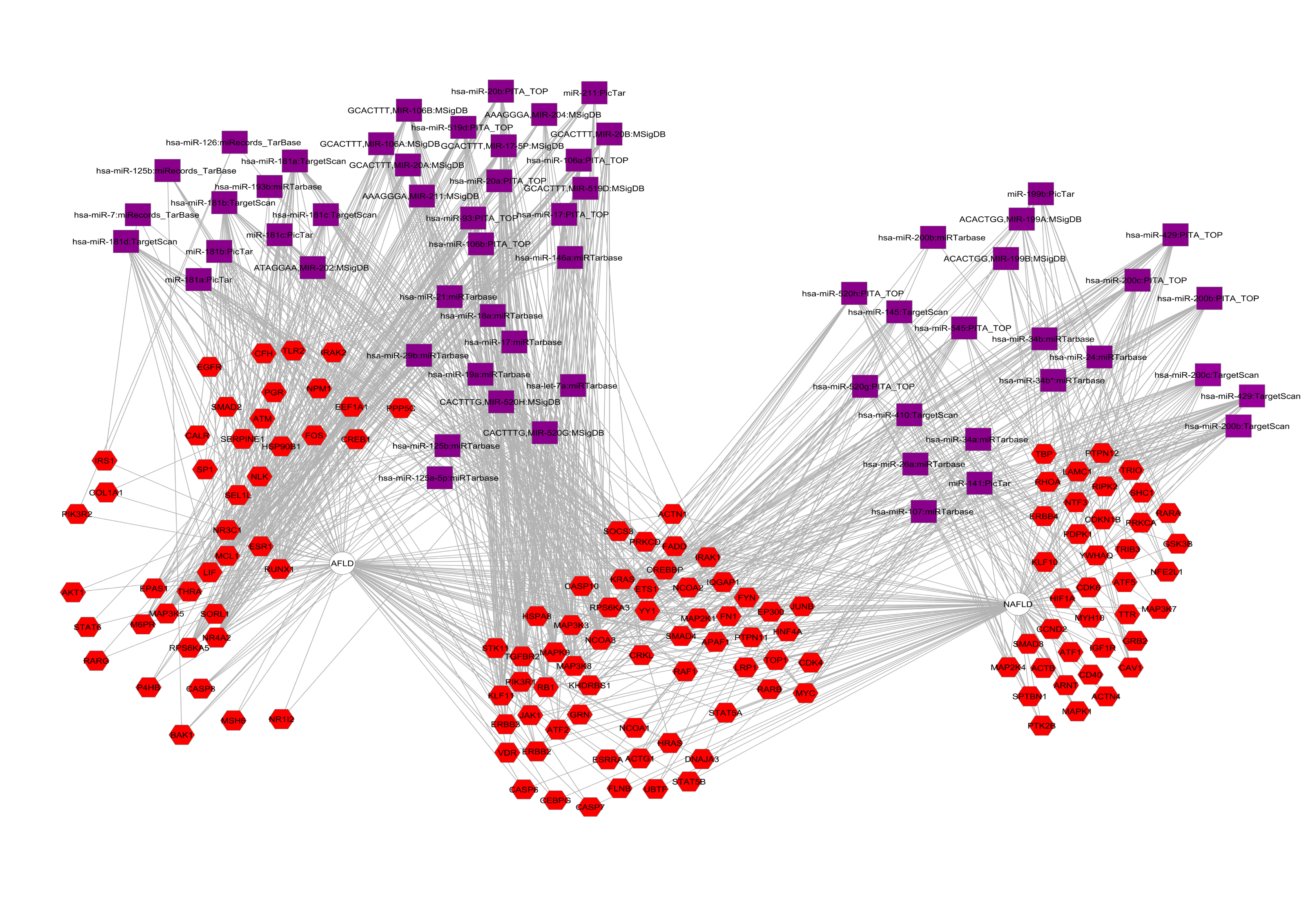

Supplement: Figure S4 — Computational prioritization of candidate genes underlying NAFLD and AFLD and comparative co-analysis of predicted miRNAs (violet squares). Prioritization was done by the bioinformatic tool ENDEAVOUR, and the figure shows the results of the cluster analysis of the first top 200 prioritized candidate genes (red squares) from the whole human genome (23.712 genes), with a significant association with the training set of NAFLD and AFLD. Functional association analysis was performed by the bioinformatics resource ToppCluster (http://toppcluster.cchmc.org). The network is shown as a cytoscape graph. (TIF) [file pone.0058895.s004.tif]

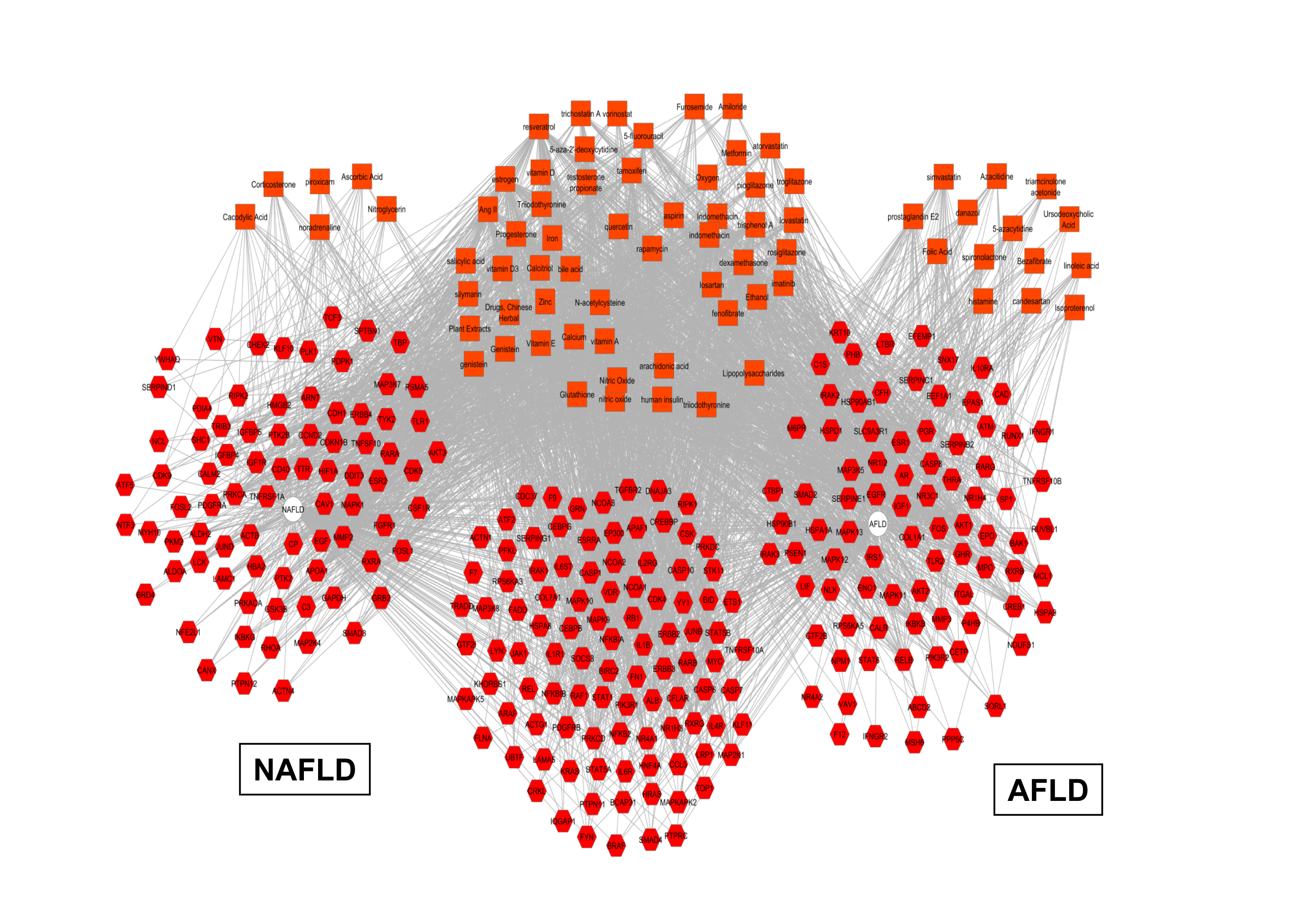

Supplement: Figure S5 — Computational prioritization of candidate genes underlying NAFLD and AFLD and comparative co-analysis of predicted drugs (orange squares). Prioritization was done by the bioinformatic tool ENDEAVOUR, and the figure shows the results of the cluster analysis of the first top 200 prioritized candidate genes (red squares) from the whole human genome (23.712 genes), with a significant association with the training set of NAFLD and AFLD. Functional association analysis was performed by the bioinformatics resource ToppCluster (http://toppcluster.cchmc.org). The network is shown as a cytoscape graph. (TIF) [file pone.0058895.s005.tif]
