# Supplementary material for: Systems Biology Elucidates Common Pathogenic Mechanisms between Nonalcoholic and Alcoholic-Fatty Liver Disease
Source: PLoS One. 2013 Mar 13;8(3):e58895. doi: 10.1371/journal.pone.0058895 (PMC3596348; doi:10.1371/journal.pone.0058895)
Supplement: Table S1 — Genes terms identified in 823 published abstracts by the PESCADOR platform (Platform for Exploration of Significant Concepts Associated to co-Occurrence Relationships) with the query “alcoholic AND (steatosis OR fatty liver) NOT (non or nonalcoholic)” for AFLD. (DOC) [file pone.0058895.s006.doc]

**Supporting Table S1**

Genes terms identified in 823 published abstracts by the PESCADOR platform (**P**latform for **E**xploration of **S**ignificant **C**oncepts **A**ssociated to co-**O**ccurrence **R**elationships) with the query “alcoholic AND (steatosis OR fatty liver) NOT (non or nonalcoholic)” for AFLD.

| **Human Symbol** | **Gene ID** |
| --- | --- |
| **HADHB** | 3032 |
| **A2M** | 2 |
| **ACACA** | 31 |
| **AVP** | 551 |
| **ADIPOQ** | 9370 |
| **ADIPOR1** | 51094 |
| **ADIPOR2** | 79602 |
| **CFD** | 1675 |
| **ALLC** | 55821 |
| **ABCD1** | 215 |
| **ALDH2** | 217 |
| **GTF2A1L** | 11036 |
| **ALPP** | 250 |
| **SERPINA1** | 5265 |
| **PRKAA1** | 5562 |
| **NOVA2** | 4858 |
| **APOA1** | 335 |
| **APOB** | 338 |
| **APOE** | 348 |
| **AQP9** | 366 |
| **ASS1** | 445 |
| **ABCB7** | 22 |
| **GOT2** | 2806 |
| **GOT1** | 2805 |
| **BAX** | 581 |
| **BCL2** | 596 |
| **BCL2L1** | 598 |
| **BECN1** | 8678 |
| **ACTB** | 60 |
| **GLB1** | 2720 |
| **HEXB** | 3074 |
| **BHMT** | 635 |
| **BTD** | 686 |
| **BRF1** | 2972 |
| **IBSP** | 3381 |
| **JUN** | 3725 |
| **MET** | 4233 |
| **DDIT3** | 1649 |
| **CA1** | 759 |
| **CA2** | 760 |
| **CA3** | 761 |
| **CA4** | 762 |
| **CNR1** | 1268 |
| **CPT1A** | 1374 |
| **CASP3** | 836 |
| **CAT** | 847 |
| **CTSB** | 1508 |
| **CNR2** | 1269 |
| **CCL4** | 6351 |
| **CCR2** | 729230 |
| **CD14** | 929 |
| **CD27** | 939 |
| **CD34** | 947 |
| **CD40** | 958 |
| **CD68** | 968 |
| **CD80** | 941 |
| **CD86** | 942 |
| **KRT18** | 3875 |
| **KRT19** | 3880 |
| **SELPLG** | 6404 |
| **COL1A2** | 1278 |
| **C3** | 718 |
| **NR1I3** | 9970 |
| **PTGS2** | 5743 |
| **CSF1** | 1435 |
| **PPIG** | 9360 |
| **CYP2B6** | 1555 |
| **CYP2E1** | 1571 |
| **DGAT1** | 8694 |
| **SLC26A3** | 1811 |
| **CDH1** | 999 |
| **E2F1** | 1869 |
| **EGR1** | 1958 |
| **EPOR** | 2057 |
| **MAPK1** | 5594 |
| **MAPK3** | 5595 |
| **ESD** | 2098 |
| **FANCA** | 2175 |
| **FAAH** | 2166 |
| **FAS** | 355 |
| **UBD** | 10537 |
| **FASN** | 2194 |
| **ADH5** | 128 |
| **FTH1** | 2495 |
| **FOXO1** | 2308 |
| **BRD2** | 6046 |
| **GAPDH** | 2597 |
| **GC** | 2638 |
| **QPCT** | 25797 |
| **GGT1** | 2678 |
| **GCK** | 2645 |
| **GSTA1** | 2938 |
| **GK** | 2710 |
| **GPAM** | 57678 |
| **GPT** | 2875 |
| **GCGR** | 2642 |
| **HSPA5** | 3309 |
| **GSTK1** | 373156 |
| **HPGDS** | 27306 |
| **HBE1** | 3046 |
| **HCC** | 619501 |
| **HGF** | 3082 |
| **HAMP** | 57817 |
| **HHEX** | 3087 |
| **HIF1A** | 3091 |
| **HMGCL** | 3155 |
| **HMGCR** | 3156 |
| **ELANE** | 1991 |
| **HMOX1** | 3162 |
| **HSBP1** | 3281 |
| **SERPINH1** | 871 |
| **HSPA1B** | 3304 |
| **HSP90AA1** | 3320 |
| **ICAM1** | 3383 |
| **IFNB1** | 3456 |
| **IFNG** | 3458 |
| **CD79A** | 973 |
| **IGF1R** | 3480 |
| **IGF2** | 3481 |
| **IGF2R** | 3482 |
| **CD40LG** | 959 |
| **IL1A** | 3552 |
| **IL10** | 3586 |
| **IL10RB** | 3588 |
| **IL13** | 3596 |
| **IL18** | 3606 |
| **IL1RN** | 3557 |
| **IL22** | 50616 |
| **IL3** | 3562 |
| **IL4** | 3565 |
| **IL6** | 3569 |
| **ISYNA1** | 51477 |
| **INSIG1** | 3638 |
| **INS** | 3630 |
| **INSR** | 3643 |
| **IRF3** | 3661 |
| **IRF7** | 3665 |
| **MAPK8** | 5599 |
| **KRT1** | 3848 |
| **KRT12** | 3859 |
| **KRT32** | 3882 |
| **FABP1** | 2168 |
| **LBP** | 3929 |
| **LDLR** | 3949 |
| **LEP** | 3952 |
| **GNRH1** | 2796 |
| **LIFR** | 3977 |
| **LPIN1** | 23175 |
| **PSMB9** | 5698 |
| **PSMB8** | 5696 |
| **LPL** | 4023 |
| **LPO** | 4025 |
| **CD46** | 4179 |
| **CCL2** | 6347 |
| **MCAT** | 27349 |
| **LY96** | 23643 |
| **MDH2** | 4191 |
| **MIP** | 4284 |
| **MMP13** | 4322 |
| **MMP2** | 4313 |
| **MMP9** | 4318 |
| **SOD2** | 6648 |
| **KLK6** | 5653 |
| **MT1B** | 4490 |
| **MYD88** | 4615 |
| **NFKB1** | 4790 |
| **NOS2** | 4843 |
| **NOS3** | 4846 |
| **SPNS1** | 83985 |
| **SPP1** | 6696 |
| **MAPK14** | 1432 |
| **TP53** | 7157 |
| **PIK3R3** | 8503 |
| **RELA** | 5970 |
| **PSIP1** | 11168 |
| **PNPLA3** | 80339 |
| **SERBP1** | 26135 |
| **PC** | 5091 |
| **PCSK9** | 255738 |
| **PADI1** | 29943 |
| **PER1** | 5187 |
| **PPARA** | 5465 |
| **PPARG** | 5468 |
| **PLA2G4A** | 5321 |
| **PIK3CA** | 5290 |
| **PLA2G6** | 8398 |
| **PLA2G2A** | 5320 |
| **PLG** | 5340 |
| **PRL** | 5617 |
| **PCNA** | 5111 |
| **PTEN** | 5728 |
| **AMACR** | 23600 |
| **RARA** | 5914 |
| **RXRA** | 6256 |
| **RPS6KA1** | 6195 |
| **S1PR1** | 1901 |
| **ACSM3** | 6296 |
| **SCAP** | 22937 |
| **SIRT1** | 23411 |
| **SMAD3** | 4088 |
| **SMAD7** | 4092 |
| **SOD1** | 6647 |
| **SST** | 6750 |
| **SREBF2** | 6721 |
| **ST6GAL1** | 6480 |
| **STAT3** | 6774 |
| **SCD** | 6319 |
| **SREBF1** | 6720 |
| **TAP1** | 6890 |
| **SERPINA7** | 6906 |
| **TBP** | 6908 |
| **TFG** | 10342 |
| **TG** | 7038 |
| **TGFB1** | 7040 |
| **WAS** | 7454 |
| **TIMP1** | 7076 |
| **TLR4** | 7099 |
| **TNF** | 7124 |
| **TNFRSF1A** | 7132 |
| **TNFRSF1B** | 7133 |
| **TRH** | 7200 |
| **TICAM1** | 148022 |
| **TXN** | 7295 |
| **UGT1A1** | 54658 |
| **UGT1A9** | 54600 |
| **UCK2** | 7371 |
| **SLC27A2** | 11001 |
| **TJP1** | 7082 |
