# Supplementary material for: Systems Biology Elucidates Common Pathogenic Mechanisms between Nonalcoholic and Alcoholic-Fatty Liver Disease
Source: PLoS One. 2013 Mar 13;8(3):e58895. doi: 10.1371/journal.pone.0058895 (PMC3596348; doi:10.1371/journal.pone.0058895)
Supplement: Table S2 — Genes terms identified in 868 published abstracts by the PESCADOR platform (Platform for Exploration of Significant Concepts Associated to co-Occurrence Relationships) with the query “nonalcoholic OR non-alcoholic) AND (fatty liver OR steatosis)” for NAFLD. (DOC) [file pone.0058895.s007.doc]

**Supporting Table S2**

Genes terms identified in 868 published abstracts by the PESCADOR platform (**P**latform for **E**xploration of **S**ignificant **C**oncepts **A**ssociated to co-**O**ccurrence **R**elationships) with the query “nonalcoholic OR non-alcoholic) AND (fatty liver OR steatosis)” for NAFLD.

| **Human Symbol** | **Gene ID** |
| --- | --- |
| **HMGCR** | 3156 |
| **ALOX5** | 240 |
| **DDC** | 1644 |
| **ABCA1** | 19 |
| **ABCB1** | 5243 |
| **ABCC1** | 4363 |
| **ABCC2** | 1244 |
| **ABCC3** | 8714 |
| **ABCG2** | 9429 |
| **ACACA** | 31 |
| **ACE** | 1636 |
| **ACHE** | 43 |
| **KLK15** | 55554 |
| **ACCS** | 84680 |
| **ACSL1** | 2180 |
| **ADC** | 113451 |
| **ADIPOQ** | 9370 |
| **PNPLA3** | 80339 |
| **ADIPOR1** | 51094 |
| **ADIPOR2** | 79602 |
| **CFD** | 1675 |
| **PLIN2** | 123 |
| **F8** | 2157 |
| **AHSG** | 197 |
| **AIFM1** | 9131 |
| **AKT1** | 207 |
| **AKT2** | 208 |
| **AKR1B1** | 231 |
| **ALK** | 238 |
| **ALMS1** | 7840 |
| **ALPP** | 250 |
| **SERPINA1** | 5265 |
| **PRKAA1** | 5562 |
| **AR** | 367 |
| **ANGPTL3** | 27329 |
| **NOVA2** | 4858 |
| **APOA5** | 116519 |
| **APOB** | 338 |
| **APOC3** | 345 |
| **APOE** | 348 |
| **APOBR** | 55911 |
| **CYP19A1** | 1588 |
| **AHR** | 196 |
| **MAP3K5** | 4217 |
| **SMPD1** | 6609 |
| **GOT1** | 2805 |
| **GOT2** | 2806 |
| **ATF4** | 468 |
| **ATF6** | 22926 |
| **PNPLA2** | 57104 |
| **ATP1A1** | 476 |
| **NDUFB3** | 4709 |
| **BAX** | 581 |
| **BCL2** | 596 |
| **BDNF** | 627 |
| **BLMH** | 642 |
| **BIRC3** | 330 |
| **DST** | 667 |
| **CXCR1** | 3577 |
| **JUN** | 3725 |
| **MET** | 4233 |
| **CRP** | 1401 |
| **CALM1** | 801 |
| **CALR** | 811 |
| **CPT1A** | 1374 |
| **CASP2** | 835 |
| **CASP3** | 836 |
| **CASP8** | 841 |
| **CAT** | 847 |
| **CCL2** | 6347 |
| **CCL4** | 6351 |
| **CCR2** | 729230 |
| **CD14** | 929 |
| **CD36** | 948 |
| **CD68** | 968 |
| **CDC42** | 998 |
| **CDIPT** | 10423 |
| **CDKN1A** | 1026 |
| **CHRD** | 8646 |
| **MLXIPL** | 51085 |
| **KRT18** | 3875 |
| **KRT19** | 3880 |
| **KRT7** | 3855 |
| **CLDN1** | 9076 |
| **LMF1** | 64788 |
| **CLOCK** | 9575 |
| **CMKLR1** | 1240 |
| **BCR** | 613 |
| **CTGF** | 1490 |
| **PTGS2** | 5743 |
| **CREB1** | 1385 |
| **CREB3L3** | 84699 |
| **CORIN** | 10699 |
| **NLRP3** | 114548 |
| **CSF2** | 1437 |
| **CXCL10** | 3627 |
| **CYP1A2** | 1544 |
| **CYP2E1** | 1571 |
| **CYCS** | 54205 |
| **DBP** | 1628 |
| **TNFRSF10B** | 8795 |
| **DGAT2** | 84649 |
| **DLC1** | 10395 |
| **DPP4** | 1803 |
| **ALDH1A1** | 216 |
| **E2F1** | 1869 |
| **EGFR** | 1956 |
| **ELOVL6** | 79071 |
| **ETFA** | 2108 |
| **EDN1** | 1906 |
| **EPHB2** | 2048 |
| **ESR1** | 2099 |
| **TNFRSF1B** | 7133 |
| **XPO1** | 7514 |
| **FAS** | 355 |
| **FASLG** | 356 |
| **FASN** | 2194 |
| **SLC27A2** | 11001 |
| **SLC27A4** | 10999 |
| **CFB** | 629 |
| **FBP1** | 2203 |
| **FGF21** | 26291 |
| **FOS** | 2353 |
| **FOXA1** | 3169 |
| **FOXA2** | 3170 |
| **FOXP3** | 50943 |
| **BRD2** | 6046 |
| **NR1H4** | 9971 |
| **CSF3** | 1440 |
| **G6PC** | 2538 |
| **GC** | 2638 |
| **GCK** | 2645 |
| **GCKR** | 2646 |
| **GCLC** | 2729 |
| **RAPGEF5** | 9771 |
| **GGT1** | 2678 |
| **GHSR** | 2693 |
| **GHR** | 2690 |
| **GHRL** | 51738 |
| **GLI2** | 2736 |
| **GCG** | 2641 |
| **GLP1R** | 2740 |
| **NR3C1** | 2908 |
| **GSTA1** | 2938 |
| **GSTT1** | 2952 |
| **GPAM** | 57678 |
| **HSPA5** | 3309 |
| **GPT** | 2875 |
| **HSP90B1** | 7184 |
| **GSTM1** | 2944 |
| **GSTP1** | 2950 |
| **HPGDS** | 27306 |
| **HP** | 3240 |
| **HBA1** | 3039 |
| **HBB** | 3043 |
| **C5** | 727 |
| **HCC** | 619501 |
| **HCCS** | 3052 |
| **HDAC3** | 8841 |
| **HAMP** | 57817 |
| **HFE** | 3077 |
| **HMGB1** | 3146 |
| **HMOX1** | 3162 |
| **HR** | 55806 |
| **ICAM1** | 3383 |
| **CD79A** | 973 |
| **IGF1** | 3479 |
| **CD40LG** | 959 |
| **IKBKB** | 3551 |
| **IL10** | 3586 |
| **IL17A** | 3605 |
| **IL18** | 3606 |
| **IL21** | 59067 |
| **IL4** | 3565 |
| **IL6** | 3569 |
| **IL7** | 3574 |
| **IL8** | 3576 |
| **IL1A** | 3552 |
| **IL1RN** | 3557 |
| **IL3** | 3562 |
| **NOS3** | 4846 |
| **NOS2** | 4843 |
| **INS** | 3630 |
| **INSR** | 3643 |
| **IRS1** | 3667 |
| **IRS2** | 8660 |
| **IDE** | 3416 |
| **JAK2** | 3717 |
| **MAPK8** | 5599 |
| **TBCE** | 6905 |
| **KLF6** | 1316 |
| **FABP1** | 2168 |
| **LAMA1** | 284217 |
| **LDLR** | 3949 |
| **LEP** | 3952 |
| **LEPR** | 3953 |
| **LTF** | 4057 |
| **LIF** | 3976 |
| **OLR1** | 4973 |
| **PLA2G7** | 7941 |
| **LPO** | 4025 |
| **MTOR** | 2475 |
| **MAT1A** | 4143 |
| **MAVS** | 57506 |
| **ABR** | 29 |
| **TFB1M** | 51106 |
| **TFB2M** | 64216 |
| **MAP3K11** | 4296 |
| **MMP9** | 4318 |
| **MMP1** | 4312 |
| **MOGAT1** | 116255 |
| **MOGAT2** | 80168 |
| **MOGAT3** | 346606 |
| **MPO** | 4353 |
| **C7orf49** | 78996 |
| **MSR1** | 4481 |
| **MTHFR** | 4524 |
| **MTTP** | 4547 |
| **MYD88** | 4615 |
| **NCAN** | 1463 |
| **NUCB2** | 4925 |
| **NFKB1** | 4790 |
| **NGF** | 4803 |
| **NLRP6** | 171389 |
| **NOS1** | 4842 |
| **NRF1** | 4899 |
| **NFE2L2** | 4780 |
| **OATP1** | 4945 |
| **OCLN** | 4950 |
| **OSM** | 5008 |
| **BGLAP** | 632 |
| **SPP1** | 6696 |
| **MAPK14** | 1432 |
| **TP53** | 7157 |
| **SQSTM1** | 8878 |
| **RELA** | 5970 |
| **PIK3R2** | 5296 |
| **SERPINE1** | 5054 |
| **PANX1** | 24145 |
| **F2R** | 2149 |
| **PCNA** | 5111 |
| **PCSK9** | 255738 |
| **PCK1** | 5105 |
| **PER1** | 5187 |
| **PPARA** | 5465 |
| **PPARG** | 5468 |
| **PEMT** | 10400 |
| **PLA2G4A** | 5321 |
| **PIK3CA** | 5290 |
| **PNO1** | 56902 |
| **PON1** | 5444 |
| **PPP1R3B** | 79660 |
| **NR1I2** | 8856 |
| **PGR** | 5241 |
| **PSMA7** | 5688 |
| **PTEN** | 5728 |
| **PTMS** | 5763 |
| **PTPN1** | 5770 |
| **PTCHD3** | 374308 |
| **PKLR** | 5313 |
| **RAC1** | 5879 |
| **AMACR** | 23600 |
| **RPTOR** | 57521 |
| **RBP4** | 5950 |
| **RIPK3** | 11035 |
| **RETN** | 56729 |
| **TSPAN31** | 6302 |
| **SCD** | 6319 |
| **B4GALNT2** | 124872 |
| **SLC6A4** | 6532 |
| **SHBG** | 6462 |
| **SIRT1** | 23411 |
| **SMAD2** | 4087 |
| **RGN** | 9104 |
| **SOD1** | 6647 |
| **SOD2** | 6648 |
| **SP1** | 6667 |
| **PLA2G10** | 8399 |
| **SREBF1** | 6720 |
| **SREBF2** | 6721 |
| **STAT3** | 6774 |
| **STAT6** | 6778 |
| **ITSN2** | 50618 |
| **CDH9** | 1007 |
| **TAP1** | 6890 |
| **TES** | 26136 |
| **TF** | 7018 |
| **TFAM** | 7019 |
| **TG** | 7038 |
| **TGM2** | 7052 |
| **TGFB1** | 7040 |
| **TH1L** | 51497 |
| **THRA** | 7067 |
| **TIMP1** | 7076 |
| **TIMP3** | 7078 |
| **PLIN3** | 10226 |
| **TLR2** | 7097 |
| **TLR4** | 7099 |
| **TLR9** | 54106 |
| **TNF** | 7124 |
| **NCRNA00273** | 649159 |
| **TCF4** | 6925 |
| **UCP1** | 7350 |
| **UCP2** | 7351 |
| **UGCG** | 7357 |
| **UCK2** | 7371 |
| **SERPINA12** | 145264 |
| **VIM** | 7431 |
| **XBP1** | 7494 |
| **CHI3L1** | 1116 |
| **ZNF267** | 10308 |
| **IL23A** | 51561 |
| **CD46** | 4179 |
| **CASP1** | 834 |
