# Supplementary material for: Systems Biology Elucidates Common Pathogenic Mechanisms between Nonalcoholic and Alcoholic-Fatty Liver Disease
Source: PLoS One. 2013 Mar 13;8(3):e58895. doi: 10.1371/journal.pone.0058895 (PMC3596348; doi:10.1371/journal.pone.0058895)
Supplement: Table S3 — Results of gene prioritization: the top ranked candidate genes prioritized for NAFLD. (DOC) [file pone.0058895.s008.doc]

**Supporting** T**able S3**

Results of gene prioritization: the top ranked candidate genes prioritized for NAFLD

| **Gene** | **Name** | **P-val** | **Rank ratio** | **Annotation EnsemblEst** |
| --- | --- | --- | --- | --- |
| ENSG00000005339 | CREBBP | 3.33E-09 | 4.44E-05 | 2.23E-148 |
| ENSG00000140443 | IGF1R | 3.76E-08 | 8.88E-05 | 9.88E-125 |
| ENSG00000135446 | CDK4 | 3.96E-08 | 0.00013326 | 1.59E-152 |
| ENSG00000145675 | PIK3R1 | 1.54E-07 | 0.00022209 | 2.83E-144 |
| ENSG00000067182 | TNFRSF1A | 1.72E-07 | 0.00026651 | 5.07E-142 |
| ENSG00000077150 | NFKB2 | 1.74E-07 | 0.00031093 | 1.20E-125 |
| ENSG00000136997 | MYC | 1.77E-07 | 0.00035535 | 1.14E-158 |
| ENSG00000100393 | EP300 | 2.23E-07 | 0.00039977 | 8.88E-135 |
| ENSG00000172216 | CEBPB | 2.75E-07 | 0.00044419 | 7.73E-143 |
| ENSG00000113721 | PDGFRB | 3.34E-07 | 0.00048861 | 2.57E-146 |
| ENSG00000111424 | VDR | 4.23E-07 | 0.00053303 | 5.87E-118 |
| ENSG00000101076 | HNF4A | 4.26E-07 | 0.00057744 | 5.70E-78 |
| ENSG00000186350 | RXRA | 4.59E-07 | 0.00062186 | 4.19E-143 |
| ENSG00000065361 | ERBB3 | 5.55E-07 | 0.00066628 | 1.19E-143 |
| ENSG00000138794 | CASP6 | 6.15E-07 | 0.0007107 | 1.56E-96 |
| ENSG00000115414 | FN1 | 7.54E-07 | 0.00075512 | 7.00E-128 |
| ENSG00000163513 | TGFBR2 | 7.80E-07 | 0.00079954 | 2.53E-153 |
| ENSG00000100030 | MAPK1 | 9.70E-07 | 0.00084396 | 3.02E-151 |
| ENSG00000169032 | MAP2K1 | 1.18E-06 | 0.00088838 | 5.66E-150 |
| ENSG00000115966 | ATF2 | 1.28E-06 | 0.00093279 | 1.06E-138 |
| ENSG00000196924 | FLNA | 1.30E-06 | 0.00097721 | 1.03E-142 |
| ENSG00000003400 | CASP10 | 2.00E-06 | 0.00111047 | 3.99E-130 |
| ENSG00000117020 | AKT3 | 2.00E-06 | 0.00115489 | 1.54E-138 |
| ENSG00000140575 | IQGAP1 | 2.63E-06 | 0.00119931 | 6.47E-157 |
| ENSG00000125730 | C3 | 2.69E-06 | 0.00124373 | 3.50E-134 |
| ENSG00000188536 | HBA2 | 2.80E-06 | 0.00128814 | 7.71E-127 |
| ENSG00000121774 | KHDRBS1 | 2.86E-06 | 0.00133256 | 1.05E-155 |
| ENSG00000132155 | RAF1 | 3.12E-06 | 0.00137698 | 2.67E-149 |
| ENSG00000137752 | CASP1 | 3.16E-06 | 0.0014214 | 6.33E-119 |
| ENSG00000179295 | PTPN11 | 3.22E-06 | 0.00146582 | 5.26E-145 |
| ENSG00000175197 | DDIT3 | 3.28E-06 | 0.00151024 | 4.69E-143 |
| ENSG00000038382 | TRIO | 3.93E-06 | 0.00155466 | 4.08E-150 |
| ENSG00000162924 | REL | 4.02E-06 | 0.00159908 | 3.69E-79 |
| ENSG00000124151 | NCOA3 | 4.04E-06 | 0.00164349 | 2.15E-134 |
| ENSG00000025434 | NR1H3 | 4.11E-06 | 0.00168791 | 7.76E-148 |
| ENSG00000141736 | ERBB2 | 4.32E-06 | 0.00173233 | 1.40E-148 |
| ENSG00000130702 | LAMA5 | 4.40E-06 | 0.00177675 | 5.18E-133 |
| ENSG00000185652 | NTF3 | 4.63E-06 | 0.00182117 | 1.04E-37 |
| ENSG00000118137 | APOA1 | 4.70E-06 | 0.00186559 | 2.79E-92 |
| ENSG00000110330 | BIRC2 | 4.75E-06 | 0.00191001 | 7.17E-150 |
| ENSG00000105639 | INSL3|JAK3 | 4.78E-06 | 0.00195443 | 1.16E-38 |
| ENSG00000115415 | STAT1 | 5.23E-06 | 0.00199885 | 8.28E-137 |
| ENSG00000003402 | CFLAR | 5.25E-06 | 0.00204326 | 2.96E-136 |
| ENSG00000077238 | IL4R | 5.26E-06 | 0.00208768 | 3.34E-150 |
| ENSG00000166285 | NO ID | 5.35E-06 | 0.0021321 | 7.10E-135 |
| ENSG00000166949 | SMAD3 | 5.73E-06 | 0.00217652 | 4.62E-145 |
| ENSG00000143933 | CALM2 | 6.05E-06 | 0.00222094 | 6.45E-145 |
| ENSG00000100906 | NFKBIA | 6.27E-06 | 0.00226536 | 4.86E-155 |
| ENSG00000078061 | ARAF | 6.33E-06 | 0.00230978 | 1.73E-153 |
| ENSG00000105401 | CDC37 | 6.41E-06 | 0.0023542 | 8.40E-144 |
| ENSG00000105397 | TYK2 | 6.52E-06 | 0.00239861 | 2.12E-149 |
| ENSG00000171223 | JUNB | 7.66E-06 | 0.00244303 | 1.51E-137 |
| ENSG00000135862 | LAMC1 | 8.46E-06 | 0.00248745 | 3.66E-150 |
| ENSG00000103653 | CSK | 8.52E-06 | 0.00253187 | 1.29E-136 |
| ENSG00000115461 | IGFBP5 | 8.57E-06 | 0.00257629 | 1.45E-140 |
| ENSG00000160014 | NO ID | 8.74E-06 | 0.00262071 | 1.16E-151 |
| ENSG00000161570 | CCL5 | 9.47E-06 | 0.00266513 | 3.93E-113 |
| ENSG00000010810 | FYN | 9.57E-06 | 0.00270955 | 9.97E-148 |
| ENSG00000123268 | ATF1 | 9.63E-06 | 0.00275396 | 2.23E-139 |
| ENSG00000139687 | RB1 | 9.71E-06 | 0.00279838 | 7.90E-146 |
| ENSG00000115594 | IL1R1 | 1.02E-05 | 0.0028428 | 6.21E-144 |
| ENSG00000165806 | CASP7 | 1.09E-05 | 0.00293164 | 2.71E-127 |
| ENSG00000198909 | MAP3K3 | 1.10E-05 | 0.00297606 | 7.21E-134 |
| ENSG00000057593 | F7 | 1.12E-05 | 0.00302048 | 5.48E-72 |
| ENSG00000109971 | HSPA8 | 1.14E-05 | 0.0030649 | 6.33E-147 |
| ENSG00000134352 | IL6ST | 1.23E-05 | 0.00310931 | 2.02E-125 |
| ENSG00000134954 | ETS1 | 1.25E-05 | 0.00315373 | 1.70E-155 |
| ENSG00000102871 | TRADD | 1.26E-05 | 0.00319815 | 8.65E-111 |
| ENSG00000133703 | KRAS | 1.27E-05 | 0.00324257 | 6.51E-152 |
| ENSG00000131759 | RARA | 1.38E-05 | 0.00328699 | 1.27E-131 |
| ENSG00000101017 | CD40 | 1.40E-05 | 0.00333141 | 2.38E-88 |
| ENSG00000082701 | GSK3B | 1.41E-05 | 0.00337583 | 5.67E-98 |
| ENSG00000084676 | NCOA1 | 1.43E-05 | 0.00342025 | 9.90E-140 |
| ENSG00000105810 | CDK6 | 1.45E-05 | 0.00346466 | 1.89E-148 |
| ENSG00000081237 | PTPRC | 1.45E-05 | 0.00350908 | 1.90E-146 |
| ENSG00000154229 | PRKCA | 1.45E-05 | 0.0035535 | 5.38E-141 |
| ENSG00000075426 | FOSL2 | 1.49E-05 | 0.00359792 | 3.26E-156 |
| ENSG00000162434 | JAK1 | 1.51E-05 | 0.00364234 | 4.45E-151 |
| ENSG00000173153 | ESRRA | 1.59E-05 | 0.00368676 | 1.35E-151 |
| ENSG00000100811 | YY1 | 1.61E-05 | 0.00373118 | 1.52E-136 |
| ENSG00000050748 | MAPK9 | 1.68E-05 | 0.0037756 | 3.50E-132 |
| ENSG00000140009 | ESR2 | 1.78E-05 | 0.00382002 | 5.81E-11 |
| ENSG00000123358 | NR4A1 | 1.80E-05 | 0.00386443 | 2.64E-145 |
| ENSG00000127022 | CANX | 1.82E-05 | 0.00390885 | 9.83E-150 |
| ENSG00000178209 | PLEC1 | 1.84E-05 | 0.00395327 | 1.06E-147 |
| ENSG00000174775 | HRAS | 1.85E-05 | 0.00399769 | 8.82E-129 |
| ENSG00000109339 | MAPK10 | 1.85E-05 | 0.00404211 | 7.49E-71 |
| ENSG00000111640 | GAPDH | 1.85E-05 | 0.00408653 | 4.75E-112 |
| ENSG00000118971 | CCND2 | 1.88E-05 | 0.00413095 | 2.59E-147 |
| ENSG00000148672 | GLUD1|GLUDP5 | 1.88E-05 | 0.00417537 | 1.21E-151 |
| ENSG00000072062 | PRKACA | 1.89E-05 | 0.00421978 | 4.84E-145 |
| ENSG00000147507 | LYN | 1.91E-05 | 0.0042642 | 1.59E-153 |
| ENSG00000130522 | JUND | 1.91E-05 | 0.00430862 | 4.52E-92 |
| ENSG00000137275 | RIPK1 | 1.97E-05 | 0.00435304 | 9.09E-140 |
| ENSG00000184009 | ACTG1 | 2.00E-05 | 0.00439746 | 1.50E-152 |
| ENSG00000140992 | PDPK1 | 2.04E-05 | 0.00444188 | 1.98E-131 |
| ENSG00000067225 | PKM2 | 2.08E-05 | 0.0044863 | 4.87E-145 |
| ENSG00000136068 | FLNB | 2.14E-05 | 0.00453072 | 3.51E-154 |
| ENSG00000118046 | STK11 | 2.18E-05 | 0.00457513 | 6.29E-135 |
| ENSG00000182866 | LCK | 2.29E-05 | 0.00461955 | 3.89E-114 |
| ENSG00000107968 | MAP3K8 | 2.30E-05 | 0.00466397 | 2.22E-120 |
| ENSG00000039068 | CDH1 | 2.39E-05 | 0.00470839 | 4.04E-145 |
| ENSG00000157764 | BRAF | 2.45E-05 | 0.00475281 | 5.34E-65 |
| ENSG00000133026 | MYH10 | 2.47E-05 | 0.00479723 | 4.13E-149 |
| ENSG00000073009 | IKBKG | 2.52E-05 | 0.00488607 | 2.21E-120 |
| ENSG00000175592 | FOSL1 | 2.53E-05 | 0.00493048 | 1.24E-122 |
| ENSG00000184557 | SOCS3 | 2.55E-05 | 0.0049749 | 1.78E-128 |
| ENSG00000163631 | ALB | 2.56E-05 | 0.00501932 | 4.50E-115 |
| ENSG00000169398 | PTK2 | 2.67E-05 | 0.00506374 | 1.00E-153 |
| ENSG00000111276 | CDKN1B | 2.77E-05 | 0.00510816 | 2.19E-144 |
| ENSG00000169136 | ATF5 | 2.78E-05 | 0.00515258 | 2.25E-142 |
| ENSG00000141646 | SMAD4 | 2.88E-05 | 0.005197 | 4.05E-150 |
| ENSG00000082641 | NFE2L1 | 2.88E-05 | 0.00524142 | 2.96E-153 |
| ENSG00000143106 | PSMA5 | 2.93E-05 | 0.00528583 | 4.80E-145 |
| ENSG00000168040 | FADD | 2.95E-05 | 0.00533025 | 1.02E-143 |
| ENSG00000138798 | EGF | 3.02E-05 | 0.00537467 | 1.54E-79 |
| ENSG00000125538 | IL1B | 3.03E-05 | 0.00541909 | 1.17E-125 |
| ENSG00000147168 | IL2RG | 3.03E-05 | 0.00546351 | 1.37E-130 |
| ENSG00000067560 | RHOA | 3.11E-05 | 0.00550793 | 5.09E-153 |
| ENSG00000126561 | STAT5A | 3.12E-05 | 0.00555235 | 5.84E-142 |
| ENSG00000121858 | TNFSF10 | 3.29E-05 | 0.00559677 | 7.17E-149 |
| ENSG00000077092 | RARB | 3.31E-05 | 0.00564119 | 8.50E-116 |
| ENSG00000115306 | SPTBN1 | 3.36E-05 | 0.0056856 | 6.77E-135 |
| ENSG00000174125 | TLR1 | 3.45E-05 | 0.00577444 | 9.89E-83 |
| ENSG00000103423 | DNAJA3 | 3.50E-05 | 0.00586328 | 1.27E-145 |
| ENSG00000071564 | TCF3 | 3.62E-05 | 0.0059077 | 1.92E-149 |
| ENSG00000163932 | PRKCD | 3.70E-05 | 0.00595212 | 2.89E-135 |
| ENSG00000172059 | KLF11 | 3.75E-05 | 0.00608537 | 4.23E-144 |
| ENSG00000109072 | VTN | 3.77E-05 | 0.00612979 | 2.87E-89 |
| ENSG00000072110 | ACTN1 | 3.79E-05 | 0.00617421 | 1.33E-151 |
| ENSG00000141959 | PFKL | 3.81E-05 | 0.00621863 | 6.76E-150 |
| ENSG00000149131 | SERPING1 | 3.85E-05 | 0.00626305 | 1.64E-151 |
| ENSG00000155660 | PDIA4 | 3.86E-05 | 0.00630747 | 2.67E-153 |
| ENSG00000160712 | IL6R | 3.90E-05 | 0.00635189 | 8.30E-124 |
| ENSG00000120868 | APAF1 | 3.90E-05 | 0.0063963 | 7.30E-134 |
| ENSG00000104312 | RIPK2 | 3.91E-05 | 0.00644072 | 1.61E-138 |
| ENSG00000153187 | HNRPU | 3.93E-05 | 0.00648514 | 4.17E-143 |
| ENSG00000164104 | HMGB2 | 3.99E-05 | 0.00652956 | 9.77E-143 |
| ENSG00000078140 | HIP2 | 4.11E-05 | 0.00657398 | 2.92E-149 |
| ENSG00000104825 | NFKBIB | 4.17E-05 | 0.0066184 | 8.58E-115 |
| ENSG00000111275 | ALDH2 | 4.24E-05 | 0.00666282 | 5.84E-141 |
| ENSG00000143437 | ARNT | 4.24E-05 | 0.00670724 | 4.49E-149 |
| ENSG00000123384 | LRP1 | 4.25E-05 | 0.00675165 | 7.37E-145 |
| ENSG00000089022 | MAPKAPK5 | 4.39E-05 | 0.00684049 | 4.13E-131 |
| ENSG00000108312 | UBTF | 4.41E-05 | 0.00688491 | 2.24E-145 |
| ENSG00000134853 | PDGFRA | 4.54E-05 | 0.00692933 | 7.20E-143 |
| ENSG00000140396 | NCOA2 | 4.57E-05 | 0.00697375 | 1.55E-121 |
| ENSG00000112592 | TBP | 4.63E-05 | 0.00701817 | 2.23E-146 |
| ENSG00000162889 | MAPKAPK2 | 4.68E-05 | 0.00706259 | 2.83E-152 |
| ENSG00000087245 | MMP2 | 4.71E-05 | 0.007107 | 4.87E-140 |
| ENSG00000121031 | PRKDC | 4.73E-05 | 0.00715142 | 2.85E-148 |
| ENSG00000185825 | BCAP31 | 4.75E-05 | 0.00719584 | 7.24E-149 |
| ENSG00000149925 | ALDOA | 4.84E-05 | 0.00728468 | 6.19E-137 |
| ENSG00000153879 | CEBPG | 4.92E-05 | 0.0073291 | 2.01E-146 |
| ENSG00000075624 | ACTB | 4.94E-05 | 0.00737352 | 2.66E-140 |
| ENSG00000105974 | CAV1 | 4.97E-05 | 0.00741794 | 1.23E-154 |
| ENSG00000166851 | PLK1 | 5.04E-05 | 0.00746236 | 1.86E-145 |
| ENSG00000030582 | GRN | 5.12E-05 | 0.00750677 | 1.12E-143 |
| ENSG00000141867 | BRD4 | 5.18E-05 | 0.00755119 | 3.61E-148 |
| ENSG00000155090 | KLF10 | 5.27E-05 | 0.00759561 | 2.61E-151 |
| ENSG00000178568 | ERBB4 | 5.32E-05 | 0.00764003 | 5.74E-37 |
| ENSG00000099937 | SERPIND1 | 5.37E-05 | 0.00768445 | 5.47E-63 |
| ENSG00000141753 | IGFBP4 | 5.48E-05 | 0.00772887 | 2.40E-145 |
| ENSG00000099942 | CRKL | 5.65E-05 | 0.00777329 | 5.98E-148 |
| ENSG00000173757 | STAT5B | 5.67E-05 | 0.00781771 | 4.58E-147 |
| ENSG00000198900 | TOP1 | 5.67E-05 | 0.00786212 | 4.77E-148 |
| ENSG00000100644 | HIF1A | 5.76E-05 | 0.00790654 | 8.53E-146 |
| ENSG00000055208 | MAP3K7IP2 | 5.84E-05 | 0.00795096 | 3.23E-154 |
| ENSG00000134308 | YWHAQ | 5.90E-05 | 0.00799538 | 5.19E-142 |
| ENSG00000177885 | GRB2 | 5.99E-05 | 0.0080398 | 5.55E-148 |
| ENSG00000101981 | F9 | 6.01E-05 | 0.00808422 | 1.01E-32 |
| ENSG00000047457 | CP | 6.07E-05 | 0.00812864 | 1.74E-88 |
| ENSG00000015475 | BID | 6.11E-05 | 0.00817306 | 4.98E-133 |
| ENSG00000120899 | PTK2B | 6.17E-05 | 0.00821747 | 3.95E-114 |
| ENSG00000183765 | CHEK2 | 6.23E-05 | 0.00826189 | 4.97E-94 |
| ENSG00000130402 | ACTN4 | 6.40E-05 | 0.00830631 | 1.56E-152 |
| ENSG00000143171 | RXRG | 6.63E-05 | 0.00835073 | 5.30E-29 |
| ENSG00000182578 | CSF1R | 6.81E-05 | 0.00839515 | 3.53E-144 |
| ENSG00000127947 | PTPN12 | 6.83E-05 | 0.00843957 | 1.30E-149 |
| ENSG00000198793 | FRAP1 | 6.85E-05 | 0.00848399 | 4.09E-150 |
| ENSG00000101255 | TRIB3 | 6.92E-05 | 0.00852841 | 4.91E-138 |
| ENSG00000104689 | TNFRSF10A | 7.07E-05 | 0.00857282 | 4.13E-63 |
| ENSG00000077782 | FGFR1 | 7.43E-05 | 0.00861724 | 3.15E-145 |
| ENSG00000115053 | NCL | 7.60E-05 | 0.00866166 | 1.81E-153 |
| ENSG00000077809 | GTF2I | 7.69E-05 | 0.00870608 | 2.83E-147 |
| ENSG00000118271 | TTR | 7.73E-05 | 0.0087505 | 2.23E-97 |
| ENSG00000114270 | COL7A1 | 7.78E-05 | 0.00879492 | 7.35E-125 |
| ENSG00000197561 | ELA2 | 7.83E-05 | 0.00883934 | 3.15E-56 |
| ENSG00000065559 | MAP2K4 | 7.89E-05 | 0.00888376 | 7.46E-140 |
| ENSG00000184047 | DIABLO | 7.92E-05 | 0.00892817 | 9.82E-150 |
