# Supplementary material for: Systems Biology Elucidates Common Pathogenic Mechanisms between Nonalcoholic and Alcoholic-Fatty Liver Disease
Source: PLoS One. 2013 Mar 13;8(3):e58895. doi: 10.1371/journal.pone.0058895 (PMC3596348; doi:10.1371/journal.pone.0058895)
Supplement: Table S4 — Results of gene prioritization: the top ranked candidate genes prioritized for AFLD. (DOC) [file pone.0058895.s009.doc]

**Supporting Table S4**

**Results of gene prioritization: the top ranked candidate genes prioritized for AFLD**

| **Gene** | **Name** | **P-value** | **Rank ratio** | **Annotation** **EnsemblEst** |
| --- | --- | --- | --- | --- |
| ENSG00000077150 | NFKB2 | 1.06E-08 | 4.43E-05 | 3.16E-134 |
| ENSG00000005339 | CREBBP | 1.49E-08 | 8.85E-05 | 4.30E-148 |
| ENSG00000100393 | EP300 | 2.24E-08 | 0.00013278 | 9.94E-139 |
| ENSG00000101076 | HNF4A | 8.00E-08 | 0.00017705 | 2.65E-61 |
| ENSG00000111424 | VDR | 9.25E-08 | 0.00022131 | 1.47E-119 |
| ENSG00000184009 | ACTG1 | 1.13E-07 | 0.00026557 | 2.57E-150 |
| ENSG00000162924 | REL | 1.23E-07 | 0.00030983 | 2.93E-87 |
| ENSG00000115414 | FN1 | 1.40E-07 | 0.00035409 | 2.29E-122 |
| ENSG00000142208 | AKT1 | 1.42E-07 | 0.00039835 | 4.38E-152 |
| ENSG00000145675 | PIK3R1 | 2.23E-07 | 0.00044261 | 4.36E-143 |
| ENSG00000196924 | FLNA | 3.09E-07 | 0.00048688 | 7.45E-147 |
| ENSG00000185591 | SP1 | 3.18E-07 | 0.00053114 | 1.01E-151 |
| ENSG00000050748 | MAPK9 | 3.49E-07 | 0.0005754 | 1.64E-134 |
| ENSG00000115415 | STAT1 | 5.14E-07 | 0.00070818 | 2.94E-146 |
| ENSG00000172216 | CEBPB | 5.80E-07 | 0.00075245 | 2.07E-143 |
| ENSG00000116016 | EPAS1 | 7.73E-07 | 0.00079671 | 3.82E-157 |
| ENSG00000169032 | MAP2K1 | 8.38E-07 | 0.00084097 | 5.31E-150 |
| ENSG00000124151 | NCOA3 | 1.13E-06 | 0.00088523 | 1.36E-132 |
| ENSG00000136997 | MYC | 1.21E-06 | 0.00092949 | 1.51E-155 |
| ENSG00000163513 | TGFBR2 | 1.38E-06 | 0.00097375 | 6.00E-150 |
| ENSG00000025434 | NR1H3 | 1.40E-06 | 0.00101801 | 8.70E-149 |
| ENSG00000126351 | THRA | 1.88E-06 | 0.00106228 | 7.14E-141 |
| ENSG00000175387 | SMAD2 | 1.92E-06 | 0.00110654 | 5.65E-141 |
| ENSG00000113580 | NR3C1 | 1.92E-06 | 0.0011508 | 2.51E-135 |
| ENSG00000135446 | CDK4 | 1.95E-06 | 0.00119506 | 7.58E-156 |
| ENSG00000102871 | TRADD | 2.05E-06 | 0.00123932 | 8.98E-110 |
| ENSG00000123358 | NR4A1 | 2.37E-06 | 0.00128358 | 5.74E-150 |
| ENSG00000125538 | IL1B | 2.67E-06 | 0.00132784 | 1.13E-110 |
| ENSG00000057593 | F7 | 2.71E-06 | 0.00137211 | 1.82E-57 |
| ENSG00000077238 | IL4R | 2.73E-06 | 0.00141637 | 7.58E-146 |
| ENSG00000105221 | AKT2 | 2.93E-06 | 0.00146063 | 1.17E-152 |
| ENSG00000123384 | LRP1 | 2.94E-06 | 0.00150489 | 8.75E-147 |
| ENSG00000113721 | PDGFRB | 3.04E-06 | 0.00154915 | 9.58E-144 |
| ENSG00000163631 | ALB | 3.28E-06 | 0.00159341 | 1.83E-100 |
| ENSG00000132155 | RAF1 | 3.40E-06 | 0.00163768 | 3.06E-152 |
| ENSG00000138794 | CASP6 | 3.46E-06 | 0.00168194 | 4.63E-103 |
| ENSG00000109971 | HSPA8 | 3.72E-06 | 0.0017262 | 7.89E-143 |
| ENSG00000064012 | CASP8 | 3.93E-06 | 0.00177046 | 1.11E-117 |
| ENSG00000160712 | IL6R | 4.28E-06 | 0.00185898 | 2.00E-115 |
| ENSG00000198909 | MAP3K3 | 4.41E-06 | 0.00190324 | 9.55E-144 |
| ENSG00000149131 | SERPING1 | 4.54E-06 | 0.00194751 | 8.94E-150 |
| ENSG00000141646 | SMAD4 | 4.57E-06 | 0.00199177 | 1.07E-150 |
| ENSG00000109339 | MAPK10 | 4.66E-06 | 0.00203603 | 6.79E-74 |
| ENSG00000130427 | EPO | 4.70E-06 | 0.00208029 | 3.41E-45 |
| ENSG00000185386 | MAPK11 | 4.71E-06 | 0.00212455 | 1.77E-79 |
| ENSG00000197561 | ELA2 | 4.75E-06 | 0.00216881 | 7.96E-59 |
| ENSG00000104365 | IKBKB | 4.76E-06 | 0.00221307 | 1.04E-147 |
| ENSG00000166598 | HSP90B1 | 5.06E-06 | 0.00225734 | 8.48E-148 |
| ENSG00000153879 | CEBPG | 5.11E-06 | 0.0023016 | 1.83E-144 |
| ENSG00000134352 | IL6ST | 5.49E-06 | 0.00234586 | 9.82E-120 |
| ENSG00000005381 | MPO | 5.55E-06 | 0.00239012 | 2.42E-79 |
| ENSG00000131187 | F12 | 6.02E-06 | 0.00243438 | 5.77E-82 |
| ENSG00000161570 | CCL5 | 6.62E-06 | 0.00252291 | 2.32E-102 |
| ENSG00000074800 | ENO1 | 6.71E-06 | 0.00256717 | 5.26E-144 |
| ENSG00000126561 | STAT5A | 6.81E-06 | 0.00261143 | 3.34E-143 |
| ENSG00000003402 | CFLAR | 7.31E-06 | 0.00265569 | 1.18E-143 |
| ENSG00000139687 | RB1 | 7.56E-06 | 0.00269995 | 1.70E-140 |
| ENSG00000165806 | CASP7 | 7.59E-06 | 0.00274421 | 1.21E-129 |
| ENSG00000179295 | PTPN11 | 7.88E-06 | 0.00278847 | 7.03E-143 |
| ENSG00000137752 | CASP1 | 8.52E-06 | 0.002877 | 5.81E-107 |
| ENSG00000168040 | FADD | 8.61E-06 | 0.00292126 | 3.07E-151 |
| ENSG00000103653 | CSK | 8.80E-06 | 0.00296552 | 3.16E-141 |
| ENSG00000146648 | EGFR | 8.81E-06 | 0.00300978 | 3.66E-131 |
| ENSG00000121031 | PRKDC | 9.44E-06 | 0.00305404 | 3.18E-154 |
| ENSG00000089022 | MAPKAPK5 | 9.49E-06 | 0.0030983 | 1.13E-132 |
| ENSG00000015475 | BID | 9.67E-06 | 0.00314257 | 8.79E-129 |
| ENSG00000130702 | LAMA5 | 9.88E-06 | 0.00318683 | 1.99E-135 |
| ENSG00000100906 | NFKBIA | 1.01E-05 | 0.00323109 | 1.94E-154 |
| ENSG00000186395 | KRT10 | 1.04E-05 | 0.00327535 | 1.04E-144 |
| ENSG00000113013 | HSPA9 | 1.05E-05 | 0.00331961 | 8.65E-151 |
| ENSG00000100811 | YY1 | 1.07E-05 | 0.00336387 | 9.58E-135 |
| ENSG00000105401 | CDC37 | 1.08E-05 | 0.00340814 | 2.23E-148 |
| ENSG00000103423 | DNAJA3 | 1.13E-05 | 0.0034524 | 1.71E-144 |
| ENSG00000170345 | FOS | 1.14E-05 | 0.00349666 | 1.09E-147 |
| ENSG00000090376 | IRAK3 | 1.18E-05 | 0.00358518 | 2.50E-48 |
| ENSG00000148672 | GLUD1|GLUDP5 | 1.21E-05 | 0.00362944 | 2.77E-149 |
| ENSG00000118260 | CREB1 | 1.23E-05 | 0.0036737 | 4.67E-147 |
| ENSG00000003056 | M6PR | 1.24E-05 | 0.00371797 | 8.17E-154 |
| ENSG00000110324 | IL10RA | 1.36E-05 | 0.00376223 | 8.54E-121 |
| ENSG00000188130 | MAPK12 | 1.38E-05 | 0.00380649 | 6.35E-89 |
| ENSG00000185825 | BCAP31 | 1.43E-05 | 0.00385075 | 1.65E-152 |
| ENSG00000104856 | RELB | 1.44E-05 | 0.00389501 | 4.05E-106 |
| ENSG00000163932 | PRKCD | 1.47E-05 | 0.00393927 | 1.13E-139 |
| ENSG00000153234 | NR4A2 | 1.48E-05 | 0.00398353 | 1.78E-113 |
| ENSG00000173153 | ESRRA | 1.56E-05 | 0.0040278 | 2.12E-150 |
| ENSG00000091831 | ESR1 | 1.57E-05 | 0.00407206 | 2.20E-68 |
| ENSG00000143171 | RXRG | 1.66E-05 | 0.00411632 | 7.83E-41 |
| ENSG00000147507 | LYN | 1.66E-05 | 0.00416058 | 2.15E-148 |
| ENSG00000012504 | NR1H4 | 1.69E-05 | 0.00420484 | 4.64E-54 |
| ENSG00000166888 | STAT6 | 1.73E-05 | 0.00429337 | 1.72E-145 |
| ENSG00000110330 | BIRC2 | 1.75E-05 | 0.00433763 | 4.20E-153 |
| ENSG00000081237 | PTPRC | 1.76E-05 | 0.00438189 | 7.67E-135 |
| ENSG00000003400 | CASP10 | 1.77E-05 | 0.00442615 | 2.29E-118 |
| ENSG00000159128 | IFNGR2 | 1.79E-05 | 0.00447041 | 3.71E-151 |
| ENSG00000149968 | MMP3 | 1.82E-05 | 0.00451467 | 5.85E-73 |
| ENSG00000030110 | BAK1 | 1.89E-05 | 0.0046032 | 1.24E-60 |
| ENSG00000137275 | RIPK1 | 1.90E-05 | 0.00464746 | 1.14E-139 |
| ENSG00000010810 | FYN | 1.90E-05 | 0.00469172 | 1.11E-147 |
| ENSG00000136068 | FLNB | 1.99E-05 | 0.00473598 | 5.82E-155 |
| ENSG00000133703 | KRAS | 2.00E-05 | 0.00478024 | 3.61E-149 |
| ENSG00000084676 | NCOA1 | 2.09E-05 | 0.0048245 | 8.41E-139 |
| ENSG00000169047 | IRS1 | 2.09E-05 | 0.00486876 | 7.06E-114 |
| ENSG00000134954 | ETS1 | 2.12E-05 | 0.00491303 | 2.48E-149 |
| ENSG00000080815 | PSEN1 | 2.12E-05 | 0.00495729 | 4.70E-135 |
| ENSG00000184557 | SOCS3 | 2.14E-05 | 0.00500155 | 1.21E-138 |
| ENSG00000108312 | UBTF | 2.18E-05 | 0.00504581 | 7.79E-147 |
| ENSG00000116062 | MSH6 | 2.19E-05 | 0.00509007 | 1.23E-136 |
| ENSG00000197771 | C10orf119 | 2.23E-05 | 0.00513433 | 1.74E-152 |
| ENSG00000111321 | LTBR | 2.27E-05 | 0.0051786 | 1.38E-142 |
| ENSG00000120889 | TNFRSF10B | 2.27E-05 | 0.00522286 | 2.27E-148 |
| ENSG00000141736 | ERBB2 | 2.32E-05 | 0.00526712 | 7.21E-147 |
| ENSG00000172819 | RARG | 2.40E-05 | 0.00535564 | 8.55E-133 |
| ENSG00000156711 | MAPK13 | 2.40E-05 | 0.0053999 | 3.55E-95 |
| ENSG00000141959 | PFKL | 2.44E-05 | 0.00544416 | 4.00E-152 |
| ENSG00000162889 | MAPKAPK2 | 2.45E-05 | 0.00548843 | 2.62E-158 |
| ENSG00000185624 | P4HB | 2.46E-05 | 0.00553269 | 4.24E-143 |
| ENSG00000115234 | SNX17 | 2.47E-05 | 0.00557695 | 2.87E-151 |
| ENSG00000084774 | CAD | 2.48E-05 | 0.00562121 | 7.35E-150 |
| ENSG00000121774 | KHDRBS1 | 2.55E-05 | 0.00566547 | 1.22E-152 |
| ENSG00000108821 | COL1A1 | 2.63E-05 | 0.00579826 | 1.10E-139 |
| ENSG00000130175 | PRKCSH | 2.65E-05 | 0.00584252 | 1.13E-148 |
| ENSG00000137462 | TLR2 | 2.68E-05 | 0.00588678 | 8.75E-116 |
| ENSG00000077092 | RARB | 2.69E-05 | 0.00593104 | 4.61E-113 |
| ENSG00000167085 | PHB | 2.72E-05 | 0.0059753 | 1.06E-145 |
| ENSG00000104825 | NFKBIB | 2.77E-05 | 0.00601956 | 3.42E-121 |
| ENSG00000197442 | MAP3K5 | 2.78E-05 | 0.00606383 | 2.09E-138 |
| ENSG00000087237 | CETP | 2.78E-05 | 0.00610809 | 3.86E-71 |
| ENSG00000159216 | RUNX1 | 2.80E-05 | 0.00615235 | 5.68E-142 |
| ENSG00000147168 | IL2RG | 2.82E-05 | 0.00619661 | 9.77E-132 |
| ENSG00000198900 | TOP1 | 2.89E-05 | 0.00624087 | 1.08E-156 |
| ENSG00000112964 | GHR | 2.98E-05 | 0.00628513 | 1.70E-138 |
| ENSG00000173757 | STAT5B | 2.99E-05 | 0.00632939 | 1.84E-147 |
| ENSG00000171223 | JUNB | 3.01E-05 | 0.00637366 | 3.68E-144 |
| ENSG00000072110 | ACTN1 | 3.02E-05 | 0.00641792 | 2.42E-153 |
| ENSG00000078061 | ARAF | 3.08E-05 | 0.00646218 | 1.76E-152 |
| ENSG00000116489 | CAPZA1 | 3.13E-05 | 0.00650644 | 1.44E-148 |
| ENSG00000109062 | SLC9A3R1 | 3.13E-05 | 0.0065507 | 9.23E-143 |
| ENSG00000099942 | CRKL | 3.22E-05 | 0.00659496 | 4.52E-148 |
| ENSG00000143384 | MCL1 | 3.28E-05 | 0.00663922 | 3.20E-151 |
| ENSG00000137642 | SORL1 | 3.31E-05 | 0.00668349 | 5.89E-130 |
| ENSG00000182326 | C1S | 3.33E-05 | 0.00672775 | 2.67E-159 |
| ENSG00000082175 | PGR | 3.34E-05 | 0.00677201 | 2.76E-28 |
| ENSG00000106366 | SERPINE1 | 3.37E-05 | 0.00681627 | 3.67E-120 |
| ENSG00000118046 | STK11 | 3.54E-05 | 0.00686053 | 4.35E-138 |
| ENSG00000157764 | BRAF | 3.58E-05 | 0.00690479 | 1.16E-57 |
| ENSG00000140575 | IQGAP1 | 3.59E-05 | 0.00694906 | 1.74E-155 |
| ENSG00000077809 | GTF2I | 3.59E-05 | 0.00699332 | 3.12E-141 |
| ENSG00000087095 | NLK | 3.64E-05 | 0.00703758 | 7.95E-120 |
| ENSG00000144852 | NR1I2 | 3.69E-05 | 0.00708184 | 1.74E-56 |
| ENSG00000023228 | NDUFS1 | 3.72E-05 | 0.0071261 | 7.60E-148 |
| ENSG00000011485 | PPP5C | 3.79E-05 | 0.00717036 | 3.42E-147 |
| ENSG00000115594 | IL1R1 | 3.84E-05 | 0.00721462 | 2.22E-138 |
| ENSG00000117601 | SERPINC1 | 3.84E-05 | 0.00725889 | 6.12E-30 |
| ENSG00000173208 | ABCD2 | 3.85E-05 | 0.00730315 | 5.92E-31 |
| ENSG00000162434 | JAK1 | 3.88E-05 | 0.00734741 | 4.87E-153 |
| ENSG00000141968 | VAV1 | 4.04E-05 | 0.00739167 | 2.28E-55 |
| ENSG00000181163 | NPM1 | 4.05E-05 | 0.00743593 | 9.91E-150 |
| ENSG00000156508 | EEF1A1 | 4.06E-05 | 0.00748019 | 3.16E-150 |
| ENSG00000179218 | CALR | 4.12E-05 | 0.00752445 | 3.23E-147 |
| ENSG00000149311 | ATM | 4.13E-05 | 0.00756872 | 1.82E-120 |
| ENSG00000055208 | MAP3K7IP2 | 4.14E-05 | 0.00761298 | 1.69E-152 |
| ENSG00000100784 | RPS6KA5 | 4.18E-05 | 0.00765724 | 7.25E-107 |
| ENSG00000005844 | ITGAL | 4.21E-05 | 0.0077015 | 2.23E-77 |
| ENSG00000071537 | SEL1L | 4.42E-05 | 0.00774576 | 1.99E-149 |
| ENSG00000159692 | CTBP1 | 4.51E-05 | 0.00779002 | 6.62E-146 |
| ENSG00000107968 | MAP3K8 | 4.56E-05 | 0.00783428 | 4.23E-123 |
| ENSG00000096384 | HSP90AB1 | 4.64E-05 | 0.00787855 | 2.44E-150 |
| ENSG00000000971 | CFH | 4.65E-05 | 0.00792281 | 1.01E-113 |
| ENSG00000030582 | GRN | 4.69E-05 | 0.00796707 | 3.74E-141 |
| ENSG00000172059 | KLF11 | 4.81E-05 | 0.00801133 | 8.72E-143 |
| ENSG00000115966 | ATF2 | 4.83E-05 | 0.00805559 | 1.21E-139 |
| ENSG00000174775 | HRAS | 4.88E-05 | 0.00809985 | 2.20E-134 |
| ENSG00000128342 | LIF | 4.98E-05 | 0.00814412 | 4.96E-93 |
| ENSG00000134070 | IRAK2 | 5.01E-05 | 0.00818838 | 9.89E-73 |
| ENSG00000120868 | APAF1 | 5.02E-05 | 0.00823264 | 1.45E-130 |
| ENSG00000065361 | ERBB3 | 5.18E-05 | 0.0082769 | 1.13E-136 |
| ENSG00000115380 | EFEMP1 | 5.24E-05 | 0.00832116 | 2.10E-155 |
| ENSG00000104689 | TNFRSF10A | 5.25E-05 | 0.00836542 | 1.73E-68 |
| ENSG00000101981 | F9 | 5.26E-05 | 0.00840968 | 5.68E-20 |
| ENSG00000175792 | RUVBL1 | 5.29E-05 | 0.00845395 | 1.76E-150 |
| ENSG00000140396 | NCOA2 | 5.40E-05 | 0.00854247 | 1.51E-117 |
| ENSG00000197632 | SERPINB2 | 5.48E-05 | 0.00863099 | 1.18E-87 |
| ENSG00000105647 | PIK3R2 | 5.54E-05 | 0.00867525 | 8.61E-130 |
| ENSG00000156261 | CCT8 | 5.64E-05 | 0.00871951 | 6.65E-147 |
| ENSG00000114270 | COL7A1 | 5.81E-05 | 0.00876378 | 5.02E-120 |
| ENSG00000144381 | HSPD1 | 5.86E-05 | 0.00880804 | 4.18E-140 |
| ENSG00000017427 | IGF1 | 5.96E-05 | 0.0088523 | 1.17E-142 |
